# The mediating role of chronic disease in socioeconomic inequalities in frailty: A longitudinal cohort study of older adults in Lausanne, Switzerland

**DOI:** 10.1016/j.tjfa.2026.100134

**Published:** 2026-03-20

**Authors:** Carlos de Mestral, Saman Khalatbari-Soltani, Patrick Bodenmann, Yves Henchoz, Mauricio Avendano

**Affiliations:** a Faculty of Biology and Medicine, University of Lausanne, Lausanne, Switzerland; b Sydney School of Public Health, Faculty of Medicine and Health, The University of Sydney, Sydney, New South Wales, Australia; c Department of Vulnerabilities and Social Medicine, Unisanté, Lausanne, Switzerland; d Department of Epidemiology and Health Systems, Unisanté, Lausanne, Switzerland

**Keywords:** Frailty, Socioeconomic factors, Chronic diseases, Obesity, Diabetes, Socioeconomic inequalities, Multimorbidity, Mediation

## Abstract

**Background:**

Frailty is a major public health concern in aging populations. Socioeconomic disadvantage increases the risk of frailty, yet the mechanisms underlying this association remain unclear.

**Objectives:**

To examine the mediating role of chronic diseases in the longitudinal association between socioeconomic disadvantage and frailty.

**Design:**

Population-based cohort study.

**Setting:**

Lausanne, Switzerland.

**Participants:**

4731 community-dwelling adults aged 65–70 years at recruitment (2004, 2010, and 2014), followed for up to 16 years, as part of the Lausanne Cohort 65+.

**Intervention:**

None.

**Measurements:**

Socioeconomic disadvantage was assessed using indicators of education, occupation, income, health insurance subsidy, and financial strain. Frailty was measured using the Fried phenotype (unintentional weight loss, exhaustion, low physical activity, weakness, and slow walking speed). Chronic conditions (obesity, diabetes, hypertension, cardiovascular and respiratory disease, and multimorbidity [≥2 conditions]) were assessed at baseline using standardized self-reported physician diagnoses. Counterfactual mediation using Cox proportional hazards models estimated the proportion of the socioeconomic disadvantage–frailty association mediated by each condition.

**Results:**

Socioeconomic disadvantage was associated with a 1.5–2.5-fold higher risk of incident frailty. Obesity mediated 13–55% of this association, diabetes 11–22%, and multimorbidity 21–39%, whereas hypertension, cardiovascular, and respiratory disease showed minimal or no mediation.

**Conclusions:**

Chronic diseases—particularly obesity and diabetes—partly explain the long-term impact of socioeconomic disadvantage on frailty, underscoring stark inequities in healthy aging. Early detection and management of these conditions in socioeconomically vulnerable older adults, alongside population-level prevention and efforts to address adverse socioeconomic conditions as root causes, could help reduce these inequalities.

## Introduction

As the global population ages, the proportion of individuals aged ≥65 years is rapidly increasing, making frailty a major public health challenge. Frailty, characterized by functional decline across multiple physiological systems and increased vulnerability to stressors [1], is associated with reduced quality of life, loneliness, falls, hospitalization, morbidity, and mortality, as well as increased health care costs [[1], [2], [3]]. While aging naturally involves some physiological decline, frailty represents an extreme and premature deterioration [1] influenced by physical, pathological, behavioral, psychosocial and socioeconomic factors over the lifecourse [[1], [2], [3]]. Research indicates that non-communicable diseases such as cardiovascular and chronic respiratory diseases, along with risk factors-including obesity [4], diabetes [5], and hypertension [6]-are strongly linked to an increased risk of frailty in later life. Moreover, frailty disproportionally affects socioeconomically disadvantaged individuals aged ≥65 years [7,8]. Although socioeconomic disadvantage is closely associated with chronic conditions and their risk factors [9], the extent to which these factors mediate the development and progression of frailty remains underexplored.

**Fig. 1:**
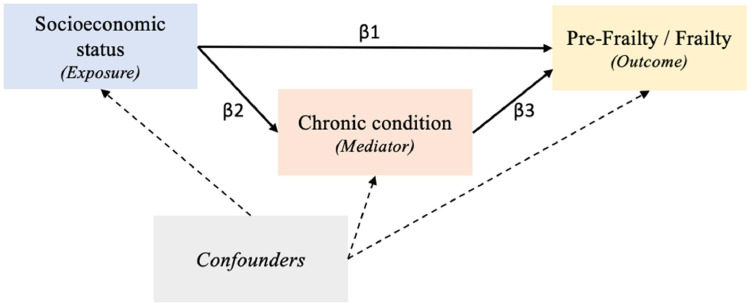
Conceptual framework representing the association between socioeconomic position, chronic conditions, and pre-frailty/frailty.

Given the modifiable nature of many chronic disease risk factors [10] and the preventability of most chronic conditions [11], understanding their mediating role could inform strategies to reduce socioeconomic inequalities in frailty and improve healthy aging outcomes. Extensive evidence indicates that socioeconomic disadvantage precedes and shapes the development of chronic diseases across adulthood, supporting the conceptualization of these conditions as mediators in this association [12,13]. This study thus aimed to assess whether chronic diseases mediate the association between socioeconomic disadvantage and frailty in older age. The conceptual framework is illustrated in Fig. 1. We used longitudinal data from the Lausanne Cohort 65+, a study of individuals aged 65–70 years at baseline and followed for up to 16 years, residing in Lausanne, Switzerland-a city of 140 000 inhabitants with highly developed educational, public transport, and healthcare infrastructure [14].

*Exposure*: Socioeconomic disadvantage as assessed by educational level, occupational position, household income level, health insurance subsidy, struggling to make ends meet, and having experience financial hardship. *Mediator*: Chronic condition including obesity, diabetes, hypertension, cardiovascular disease, chronic respiratory disease, and multimorbidity. *Potential confounders* considered in our analyses were age, sex, cohort, smoking, body mass index, and feeling isolated. According to our framework, chronic conditions are socioeconomically patterned and also associated with pre-frailty and frailty. β1 indicates the direct association of the exposure on the outcome. β2 indicates the direct association of the exposure on the mediator. β3 indicates the direct association of the mediator on the outcome.

## Methods

### Study population

The Lausanne cohort 65+ is a population-based study investigating the determinants and consequences of frailty in community-dwelling older adults in Lausanne, Switzerland. Detailed sampling and methodology are published elsewhere [15]. Briefly, 4731 individuals aged 65–70 years were randomly recruited in three waves: cohort 1 (2004), cohort 2 (2010), and cohort 3 (2014). Baseline frailty assessments were conducted within a year by trained medical assistants, followed by annual questionnaires and triennial frailty assessments. Data from cohorts were included up to their last medical assessments: cohort 1 (2005–20), cohort 2 (2010–19), and cohort 3 (2015–21). The study was approved by the Ethics Committee of the Faculty of Biology and Medicine, University of Lausanne. This study adheres to the STROBE (Strengthening the Reporting of Observational Studies in Epidemiology) guidelines (Supplementary material).

### Demographic, lifestyle, and socioeconomic indicators

Baseline variables included: sex (men vs women), age (continuous), smoking status (current vs never/former), feeling isolated (always/very frequently/frequently vs sometimes/rarely/never), Mini Nutritional Assessment score (<24: malnutrition or at risk vs ≥24: normal nutritional status) [16] and cohort (1, 2, or 3). Six socioeconomic indicators were used as baseline exposure variables: 1) Education: classified per ISCED levels into lower (basic education), middle (apprenticeship), and higher (post-compulsory education); 2) Occupational position: categorized as lower (e.g., unqualified worker), middle (e.g. qualified worker), or higher (e.g. managerial roles); and 3) Household income: reported gross monthly income was divided into tertiles: lower (<4270 CHF), middle (4270–6650 CHF), and higher (≥6651 CHF) (1 CHF ≈ 0.93 GBP as of October 13, 2025) (**Supplementary table 1**). The remaining three indicators further capture different aspects of financial disadvantage by assessing reports of: a) receipt of government health insurance subsidies (receiving partial/full subsidy vs not receiving any), b) difficulties to make ends meet (yes vs no); and c) financial hardship during previous 12 months (yes vs no).

### Chronic conditions

Five chronic conditions assessed at baseline were included: obesity (BMI ≥30 kg/m^2^ measured during examination), diabetes, hypertension, cardiovascular (CVD, including coronary heart disease, congestive heart failure, cardiopathy or valvopathy, stroke, or other CVD), and chronic respiratory disease (chronic obstructive pulmonary disease or asthma). Multimorbidity was defined as having ≥ two chronic conditions. Chronic conditions were self-reported using a standardized questionnaire assessing physician-diagnosed conditions and related medication use (**Supplementary table 1**).

### Frailty assessment

Frailty was evaluated using the Fried phenotype [2], which includes five criteria assessed via survey and medical examination: 1) unintentional weight loss; 2) exhaustion; 3) low physical activity; 4) muscular weakness; and 5) slow walking speed (**Supplementary table 1**). A frailty score was calculated as the number of criteria met, divided by number assessed, and multiplied by five [2,17]. We classified individuals as non-frail (score = 0), pre-frail (score = 1–2), and frail (score ≥3) [17].

### Statistical analysis

Participants without complete socioeconomic, demographic, chronic conditions, and frailty data were excluded from the analytical sample (**Supplementary Figure 1**). Participants were followed from baseline until the first occurrence of pre-frailty/frailty, death, loss to follow-up, or the end of the observation period (December 2021), whichever came first. To prepare for mediation analysis, we first examined the associations between the exposure (socioeconomic indicators), the mediator (chronic conditions), and the outcome (frailty and pre-frailty).

First, using baseline data only, we assessed the cross-sectional association between socioeconomic indicators and each chronic condition, with logistic regression, adjusting the estimate for age, sex, and cohort in model 1 and additionally for smoking, feeling isolated, and BMI in model 2 (except in model with obesity as dependent variable). Second, we examined the longitudinal association between socioeconomic indicators at baseline and incident frailty (and pre-frailty) over follow-up using Cox proportional hazards models. Estimates were adjusted for age, sex, and cohort in model 1, and further for smoking, BMI, and feeling of isolation in model 2. Third, we assessed the longitudinal association between each chronic condition and incident frailty (and pre-frailty) over follow-up using Cox proportional hazards models, adjusting for age, sex, and cohort in model 1, and additionally for smoking, feeling of isolation, and BMI in model 2 (except for model with obesity as the exposure).

Lastly, we examined the mediation of chronic conditions in the longitudinal association between socioeconomic disadvantage and incident frailty (and pre-frailty) using counterfactual mediation analysis [18]. This approach provides a four-way decomposition of the association between the exposure and the outcome, from which we report: 1) the total association between the exposure and the outcome (Hazard ratio and 95% CI); 2) the proportion of the association between the exposure and the outcome via pathways that include neither the mediator nor any exposure-mediator interaction (i.e., natural direct association); 3) the overall proportion of the association between the exposure and the outcome via the mediator (i.e., natural indirect association); 4) the overall proportion of the association between the exposure and the outcome via exposure-mediator interaction; and 5) the proportion eliminated, which indicates how much of the association between the exposure and the outcome could be eliminated if the mediator were set to zero [18]. In this framework, all associations involving the longitudinal outcome (frailty and pre-frailty) were modeled using Cox proportional hazards regression, whereas the association between socioeconomic indicators and each binary chronic condition (the mediator) was modeled using logistic regression. In all models, the estimates are adjusted for age, sex, and cohort in model 1, and additionally for smoking, isolation feeling, and BMI in model 2.

In sensitivity analyses, we repeated the counterfactual mediation analysis after first excluding participants with BMI <18.5 or >40, and then those classified as malnourished or at risk based on the MNA [16]. To assess the robustness of the association between socioeconomic disadvantage and incident frailty and pre-frailty, we also ran separate Cox proportional hazards models additionally adjusting for each chronic condition. Although chronic conditions were hypothesized to lie on the causal pathway, socioeconomic and chronic conditions were measured at baseline, and temporality cannot be fully ascertained; these analyses were therefore conducted as sensitivity analyses to explore potential residual confounding, acknowledging the risk of overadjustment [19]. All analyses were conducted in Stata/SE 18.0 (StataCorp, TX, USA), using the med4way command [20] for counterfactual mediation.

## Funding

From the beginning, the Lc65+ study has been financed exclusively by public funds or not-for-profit organizations. It is currently funded by the Direction générale de la santé (DGS), canton Vaud, Switzerland, the Centre for Primary Care and Public Health (Unisanté) and by a research grant from the Esther Locher-Gurtner Foundation. The funders had no role in the design, execution, analysis and interpretation of data, or writing of the study.

## Results

### Sample characteristics

Of the 4 731 participants, our analytical sample included 3 643 (77.3%) with complete data on frailty, baseline characteristics, and at least one of the socioeconomic indicators and chronic conditions. Table 1 presents the baseline characteristics. Participants were followed for a median of 7 years (IQR 4–10). Median follow-up was 13 years [[7], [8], [9], [10], [11], [12], [13], [14], [15], [16]] in cohort 1, 10 years [[7], [8], [9], [10]] in cohort 2, and 7 years [[4], [5], [6], [7]] in cohort 3. Participants' ages ranged from 66.2 to 70.8 years, and 58.6% were women. The proportion with post-compulsory education ranged from 36.5% in cohort 1 to 49.2% in cohort 3, while the proportion with higher occupational positions ranged from 34.5% in the older cohort to 44.6% in the younger cohort. Additionally, 14.0% reported receiving health insurance subsidies, 13.9% struggled to make ends meet, and 7.7% experienced financial hardship in the past year. 17.2% of participants were current smokers, and 6.5% felt isolated frequently to always. While 26.6% of participants fell into the pre-frailty group at baseline, only 1.8% were in the frailty group. Regarding chronic conditions, 22.9% had obesity, 8.9% had diabetes, 34.2% had hypertension, 10.6% had cardiovascular disease, 8.2% had chronic respiratory disease, and 22.0% had multimorbidity (Table 1).

**Table 1:** Characteristics of participants, Lausanne Cohort 65+.

	Total	Cohort 1	Cohort 2	Cohort 3
**N**	3643	1105	1230	1308
**Follow-up time** (median, IQR), y	7 (5–10)	13 (10–16)	10 (7–10)	7 (4–7)
**Age** (median, IQR), y	68 (66–70)	68 (66–70)	68 (66–70)	68 (66–70)
**Men**	1507 (41.4)	439 (39.7)	503 (40.9)	565 (43.2)
**Women**	2136 (58.6)	666 (60.3)	727 (59.1)	743 (56.8)
**Educational level**				
Basic compulsory	637 (17.6)	261 (23.8)	208 (17.0)	168 (13.0)
Apprenticeship	1422 (39.3)	436 (39.7)	496 (40.5)	490 (37.8)
Post compulsory schooling	1558 (43.1)	400 (36.5)	521 (42.5)	637 (49.2)
**Occupational position**				
Lower	681 (19.2)	254 (24.0)	240 (19.8)	187 (14.6)
Middle	1466 (41.2)	438 (41.4)	504 (41.5)	524 (40.8)
Higher	1407 (39.6)	365 (34.5)	470 (38.7)	572 (44.6)
**Household income**				
Lowest tertile	1194 (33.5)	395 (37.3)	427 (35.3)	372 (28.8)
Middle tertile	1171 (32.9)	362 (34.2)	389 (32.1)	420 (32.6)
Higher tertile	1194 (33.5)	302 (28.5)	394 (32.6)	498 (38.6)
**Receives partial/full insurance subsidy**	511 (14.0)	158 (14.3)	178 (14.5)	175 (13.4)
**Struggles making ends meet**	506 (13.9)	133 (12.0)	181 (14.7)	192 (14.7)
**Financial hardship in past year**	281 (7.7)	86 (7.8)	91 (7.4)	104 (8.0)
**Isolation feeling**				
Rarely/never	2774 (76.1)	840 (76.0)	936 (76.1)	998 (76.3)
Sometimes	634 (17.4)	177 (16.0)	224 (18.2)	233 (17.8)
Frequently/always	235 (6.5)	88 (8.0)	70 (5.7)	77 (5.9)
**Current smoker**	625 (17.2)	197 (17.8)	194 (15.8)	234 (17.9)
**Frailty score**				
Non-frailty	2518 (71.6)	799 (72.4)	844 (68.9)	875 (67.6)
Pre-frailty	933 (26.6)	254 (23.0)	332 (27.1)	347 (26.8)
Frailty	63 (1.8)	20 (1.8)	15 (1.2)	28 (2.2)
**Obesity**	833 (22.9)	262 (23.7)	290 (23.6)	281 (21.5)
**Diabetes**	314 (8.6)	86 (7.8)	120 (9.8)	108 (8.3)
**Hypertension**	1245 (34.2)	353 (32.0)	463 (37.6)	429 (32.8)
**Cardiovascular disease**	376 (10.6)	140 (13.3)	134 (11.1)	102 (7.8)
**Chronic respiratory disease**	300 (8.2)	81 (7.3)	100 (8.1)	119 (9.1)
**Multimorbidity**	803 (22.0)	241 (21.8)	297 (24.2)	265 (20.3)

Numbers are N (%) unless indicated otherwise. Occupational position categorized as higher (high managerial, middle managerial, or independent), middle (qualified employee) and lower (qualified worker/farmer, unqualified employee, unqualified laborer). IQR: interquartile range.

Participants excluded from the main analytical sample (**Supplementary figure 1**) were more socioeconomically disadvantaged across all indicators (p<0.01), with higher rates of pre-frailty (38.8% vs. 26.6%) and frailty (7.4% vs. 1.8%) (p<0.01). They also reported higher proportions of diabetes and cardiovascular disease (**Supplementary table 2**).

### Association between socioeconomic disadvantage and chronic conditions

**Fig. 2:**
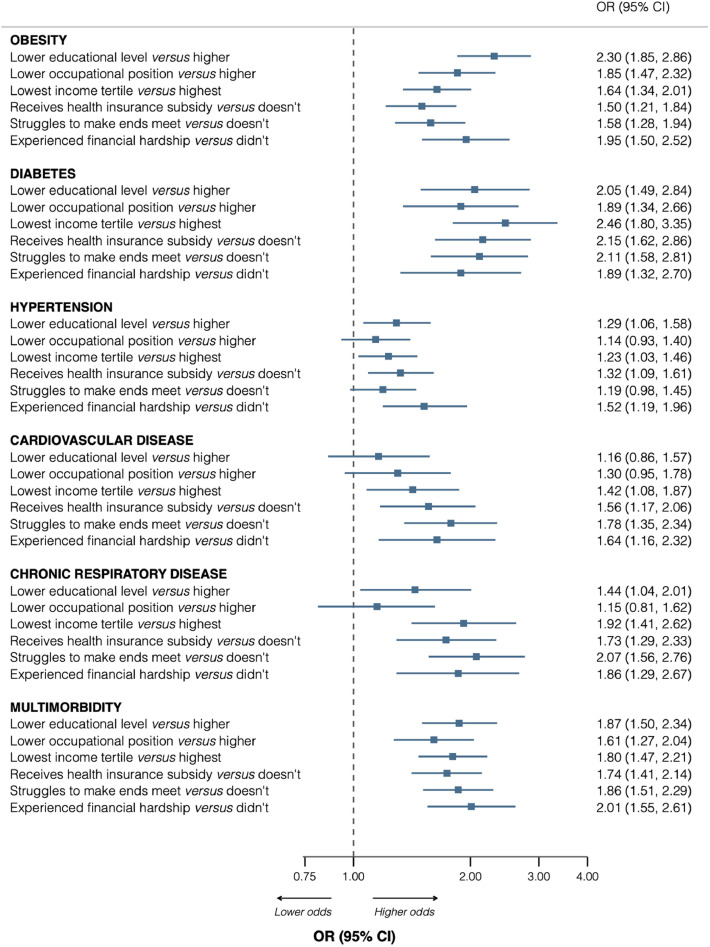
Cross-sectional association between socioeconomic disadvantage and chronic conditions at baseline, Lausanne Cohort 65+.

Fig. 2 displays odds ratios of the association between socioeconomic indicators and chronic conditions at baseline. There was a clear general pattern of association whereby socioeconomic disadvantage was associated with greater odds of having most chronic conditions. For instance, compared with higher educational level, having a lower educational level was associated with greater odds of having obesity [Odds ratio (95% CI): 2.30 (1.85–2.86)], diabetes [2.05 (1.49–2.84)], hypertension [1.29 (1.06–1.58)], respiratory disease [1.44 (1.04–2.01)], and multimorbidity [1.87 (1.50–2.34)]. Participants with a lower occupational position also had greater odds of having obesity [1.85 (1.47–2.32)], diabetes [1.89 (1.34–2.66)], and multimorbidity [1.61 (1.27–2.04)], compared with participants having a higher occupational position. Similarly, a household income in the lower tertile was associated with greater odds of having obesity [1.64 (1.34–2.01)], diabetes [2.46 (1.80–3.35)], hypertension [1.23 (1.03–1.46)], cardiovascular disease [1.42 (1.08–1.87)], respiratory disease [1.92 (1.41–2.62)], and multimorbidity [1.80 (1.47–2.21)]. Participants who reported experiencing financial hardship in the preceding year had greater odds of having obesity [1.95 (1.50–2.52)], diabetes [1.89 (1.32–2.70)], hypertension [1.52 (1.19–1.96)], cardiovascular disease [1.64 (1.16–2.32)], respiratory disease [1.86 (1.29–2.67)] and multimorbidity [2.01 (1.55–2.61)], compared with participants who did not report having any financial hardship. The pattern of association was similar for having receiving health insurance subsidy and for struggling to make ends meet (Fig. 2). Further adjustment with smoking and BMI attenuated the estimates but the pattern of association remained the same (**Supplementary figure 2**).

Results are Odds ratio estimates (95% confidence interval), adjusted for age, sex, and cohort, from Logistic regression. Multimorbidity indicates the presence of at least 2 chronic conditions. Sample size = 3643.

### Association between socioeconomic indicators and frailty

**Fig. 3:**
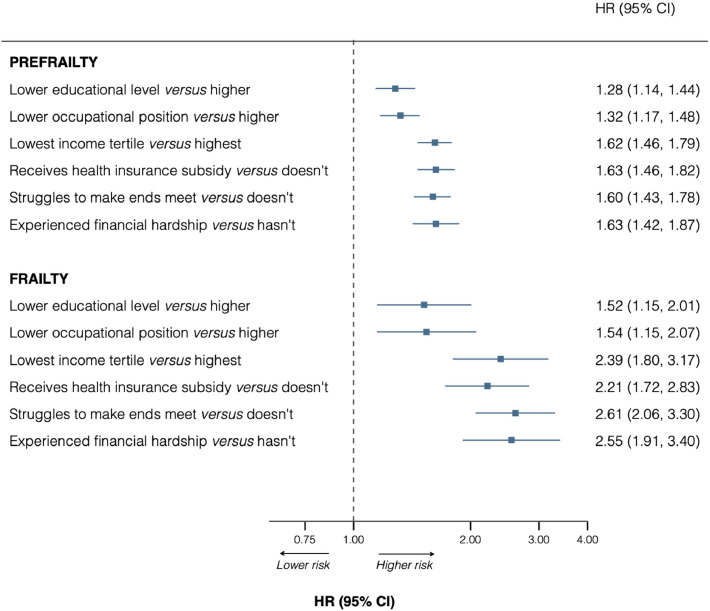
Longitudinal association between socioeconomic disadvantage at baseline and subsequent pre-frailty and frailty, Lausanne Cohort 65+.

Fig. 3 shows results from Cox Proportional Hazard models on the association between socioeconomic indicators and subsequent development of pre-frailty and frailty. Participants with lower educational level and lower occupational position had 52% and 54% greater risk of developing frailty [Hazard ratio 1.52, (95% CI: 1.15–2.01); and 1.54 (1.15–2.07), respectively] compared with the most socioeconomically advantaged participants. Participants in the lower income tertile had more than double the risk to subsequently develop frailty, compared with those in the higher income tertile [2.39 (1.80–3.17)]. Similarly, participants receiving health insurance subsidy, struggling to make ends meet, and having experienced financial hardship in the past year had more than twice the risk of developing frailty, compared to the socioeconomically advantaged participants (Fig. 3). Associations for pre-frailty were similar, with comparable gradients across socioeconomic indicators. **Supplementary figure 3** displays the results with further adjustments for smoking, BMI, and feeling of isolation; association estimates were attenuated but pattern of association remained unchanged.

Results are Hazard ratios (95% confidence interval), adjusted for age, sex, and cohort, from Cox proportional hazards regression. Sample size = 3643.

In sensitivity analysis treating chronic conditions as potential confounders rather than mediators, adjustment for individual chronic conditions attenuated—but did not eliminate—the socioeconomic gradients in frailty (**Supplementary table 3**).

### Association between chronic condition at baseline and frailty

**Fig. 4:**
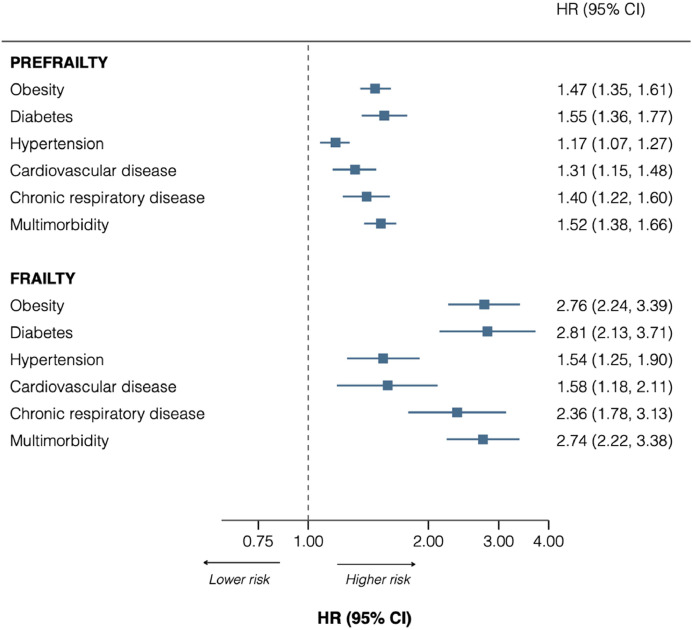
Longitudinal association between chronic condition at baseline and subsequent pre-frailty and frailty, Lausanne cohort 65+ cohort.

Fig. 4 shows hazard ratios of the association between having a chronic condition at baseline and subsequent development of frailty. Having any chronic condition at baseline was associated with higher risk of subsequent frailty, ranging from 54% higher risk for participants with hypertension [HR: 1.54 (95% CI: 1.25–1.90)] to almost three times greater risk for participants with diabetes [2.81 (2.13–3.71)], multimorbidity [2.74 (2.22–3.38)], and obesity [2.76 (2.24–3.39)] (Fig. 4). **Supplementary figure 4** displays the results with further adjustments for smoking, BMI, and feeling of isolation; results were attenuated but the pattern of associations remained unchanged.

Results are Hazard ratios (95% confidence interval), adjusted for age, sex, and cohort in Model 1, from Cox proportional hazards regression. Sample size = 3643.

### Mediation of chronic conditions in the association between socioeconomic disadvantage and frailty

Table 2 shows the results of the 4-way decomposition counterfactual mediation analysis. Socioeconomic disadvantage was consistently associated with an increased risk of developing frailty across all socioeconomic indicators. The chronic conditions that appeared to mediate the most this increased risk of frailty due to socioeconomic disadvantage were obesity, diabetes, and multimorbidity, while hypertension, cardiovascular disease, and chronic respiratory disease showed minimal, no, or non-statistically significant mediation.

**Table 2:** Mediation of chronic conditions in the association between baseline socioeconomic factors and subsequent frailty, Lausanne Cohort 65+.

Exposure	Mediator	Total association HR (95% CI)	Proportion of direct association (95% CI)	Overall proportion due to mediation (95% CI)	Overall proportion due to interaction (95% CI)	Overall portion eliminated (95% CI)
Educational level	Obesity	1.54 (1.10–1.97)	0.20 (-0.32–0.72)	0.55 (0.22–0.88)	0.47 (-0.08–1.01)	0.80 (0.28–1.32)
Occupational position		1.57 (1.09–2.04)	0.35 (-0.13–0.83)	0.39 (0.13–0.64)	0.41 (-0.10–0.92)	0.65 (0.17–1.13)
Household income		2.47 (1.77–3.16)	0.56 (0.34–0.77)	0.18 (0.09–0.27)	0.38 (0.15–0.61)	0.44 (0.23–0.66)
Health insurance subsidy		2.18 (1.63–2.72)	0.60 (0.30–0.91)	0.16 (0.05–0.27)	0.31 (0.00–0.62)	0.40 (0.09–0.70)
Struggle to make ends meet		2.47 (1.89–3.05)	0.74 (0.47–1.00)	0.13 (0.04–0.22)	0.18 (-0.09–0.46)	0.26 (0.00–0.53)
Financial hardship		2.47 (1.75–3.18)	0.37 (0.03–0.71)	0.29 (0.14–0.45)	0.52 (0.18–0.86)	0.63 (0.29–0.97)
Educational level	Diabetes	1.53 (1.10–1.95)	0.75 (0.41–1.10)	0.21 (0.01–0.41)	0.07 (-0.32–0.47)	0.25 (-0.10–0.59)
Occupational position		1.54 (1.08-2.01)	0.66 (0.29–1.03)	0.22 (0.01–0.42)	0.22 (-0.15–0.60)	0.34 (-0.03–0.71)
Household income		2.36 (1.71–3.02)	0.79 (0.62–0.95)	0.14 (0.04–0.24)	0.17 (0.01–0.34)	0.21 (0.05–0.38)
Health insurance subsidy		2.18 (1.65–2.71)	0.89 (0.67–1.11)	0.11 (0.01–0.21)	0.01 (-0.23–0.24)	0.11 (-0.11–0.33)
Struggle to make ends meet		2.64 (2.00–3.29)	0.71 (0.49–0.94)	0.17 (0.04–0.30)	0.23 (0.01–0.45)	0.29 (0.06–0.51)
Financial hardship		2.53 (1.80–3.26)	0.70 (0.43–0.97)	0.16 (0.01–0.31)	0.25 (-0.01–0.51)	0.30 (0.03–0.57)
Educational level	Hypertension	1.44 (1.02–1.85)	1.22 (0.33–2.10)	0.05 (-0.04–0.14)	-0.31 (-1.28–0.66)	-0.22 (-1.10–0.67)
Occupational position		1.50 (1.03–1.96)	0.93 (0.25–1.62)	0.04 (-0.03–0.11)	0.03 (-0.68–0.74)	0.07 (-0.62–0.75)
Household income		2.41 (1.74–3.09)	0.73 (0.46–0.99)	0.04 (0.00–0.08)	0.26 (-0.01–0.54)	0.27 (0.01–0.54)
Health insurance subsidy		2.16 (1.61–2.70)	0.87 (0.46–1.27)	0.04 (-0.02–0.10)	0.11 (-0.31–0.52)	0.13 (-0.27–0.54)
Struggle to make ends meet		2.55 (1.95–3.16)	0.81 (0.49–1.12)	0.03 (-0.01–0.07)	0.18 (-0.14–0.49)	0.19 (-0.12–0.51)
Financial hardship		2.51 (1.78–3.23)	0.78 (0.34–1.21)	0.07 (-0.02–0.15)	0.19 (-0.25–0.64)	0.22 (-0.21–0.66)
Educational level	Cardiovascular disease	1.46 (1.03–1.88)	1.11 (0.53–1.68)	0.01 (-0.04–0.06)	-0.12 (-0.71–0.47)	-0.11 (-0.68–0.47)
Occupational position		1.53 (1.04–2.02)	0.93 (0.44–1.43)	0.04 (-0.04–0.12)	0.03 (-0.50–0.56)	0.07 (-0.43–0.56)
Household income		2.65 (1.84–3.46)	0.73 (0.51–0.95)	0.07 (0.00–0.13)	0.27 (0.03–0.50)	0.27 (0.05–0.49)
Health insurance subsidy		2.29 (1.64–2.93)	0.86 (0.51–1.21)	0.06 (-0.04–0.16)	0.11 (-0.25–0.47)	0.14 (-0.21–0.49)
Struggle to make ends meet		2.67 (1.97–3.36)	0.84 (0.56–1.13)	0.07 (-0.03–0.18)	0.13 (-0.17–0.42)	0.16 (-0.13–0.44)
Financial hardship		2.45 (1.66–3.23)	1.02 (0.60–1.44)	0.02 (-0.10–0.14)	-0.06 (-0.49–0.38)	-0.02 (-0.44–0.40)
Educational level	Chronic respiratory disease	1.51 (1.09–1.94)	0.73 (0.37–1.09)	0.12 (-0.02–0.27)	0.21 (-0.16–0.58)	0.27 (-0.09–0.63)
Occupational position		1.54 (1.07–2.01)	0.80 (0.45–1.14)	0.04 (-0.07–0.15)	0.19 (-0.16–0.53)	0.20 (-0.14–0.55)
Household income		2.41 (1.74–3.08)	0.82 (0.66–0.98)	0.10 (0.02–0.18)	0.15 (-0.02–0.32)	0.18 (0.02–0.34)
Health insurance subsidy		2.21 (1.66–2.76)	0.81 (0.57–1.06)	0.10 (-0.01–0.21)	0.14 (-0.11–0.38)	0.19 (-0.06–0.43)
Struggle to make ends meet		2.58 (1.97–3.19)	0.86 (0.65–1.07)	0.10 (-0.01–0.21)	0.08 (-0.14–0.30)	0.14 (-0.07–0.35)
Financial hardship		2.46 (1.76–3.16)	0.96 (0.71–1.21)	0.05 (-0.06–0.16)	-0.02 (-0.28–0.24)	0.04 (-0.21–0.29)
Educational level	Multimorbidity	1.57 (1.12–2.02)	0.38 (-0.07–0.83)	0.39 (0.16–0.61)	0.37 (-0.16–0.90)	0.62 (0.17–1.07)
Occupational position		1.61 (1.10–2.11)	0.40 (-0.04–0.84)	0.30 (0.10–0.50)	0.42 (-0.07–0.91)	0.60 (0.16–1.04)
Household income		2.56 (1.83–3.28)	0.40 (0.19–0.61)	0.24 (0.15–0.34)	0.55 (0.33–0.78)	0.60 (0.39–0.81)
Health insurance subsidy		2.18 (1.62–2.74)	0.58 (0.26–0.89)	0.21 (0.09–0.33)	0.31 (-0.03–0.64)	0.42 (0.11–0.74)
Struggle to make ends meet		2.59 (1.97–3.22)	0.56 (0.30–0.82)	0.21 (0.11–0.31)	0.34 (0.07–0.61)	0.44 (0.18–0.70)
Financial hardship		2.47 (1.75–3.18)	0.56 (0.20–0.92)	0.23 (0.09–0.38)	0.32 (-0.05–0.69)	0.44 (0.08–0.80)

Results are from counterfactual mediation models. Multimorbidity indicates having at least two chronic conditions (diabetes, hypertension, cardiovascular disease, respiratory disease). The total association is Hazard ratio (95% CI) from Cox proportional hazard ratio, adjusted for baseline age, sex, and cohort. Proportion of direct association (95% CI) indicates the association between the exposure and the outcome that includes neither interaction nor mediation. Proportion due to mediation indicates the indirect association of the exposure with the outcome that involves pathways through the mediator. Proportion due to interaction indicates the association of the exposure with the outcomes via interaction with the mediator. Eliminated proportion indicates the proportion of the association that would be eliminated if the mediator were removed (e.g., fixed to 0). Sample size = 3643.

In the models including obesity as the mediator, the risk of developing frailty was (Hazard ratio, 95% CI) 1.54 (1.10–1.97) for individuals with lower educational level, 1.57 (1.09–2.04) for those with lower occupational position, 2.47 (1.77–3.16) for those with lower household income, 2.18 (1.63–2.72) for those receiving health insurance subsidy, 2.47 (1.89–3.05) for those struggling to make ends meet, and 2.47 (1.75–3.18) for those having experienced financial hardship. Of these associations, the proportion due to mediation was 0.55 (0.22–0.88) for educational level, 0.39 (0.13–0.64) for occupational position, 0.18 (0.09–0.27) for income level, 0.16 (0.05–0.27) for health insurance subsidy, 0.13 (0.04–0.22) for struggling to make ends meet, and 0.29 (0.14–0.45) for financial hardship (Table 2).

In the models considering diabetes as the mediator, the risk of developing frailty was 1.53 (1.10–1.95) for individuals with lower educational level, 1.54 (1.08–2.01) for those with lower occupational position, 2.36 (1.71–3.02) for those with lower household income, 2.18 (1.65–2.74) for those receiving health insurance subsidy, 2.64 (2.00–3.29) for those struggling to make ends meet, and 2.53 (1.80–3.26) for individuals having experienced financial hardship. Of these associations, the proportion mediated by diabetes was 0.21 (0.01–0.41) for educational level, 0.22 (0.02–0.42) for occupational position, 0.14 (0.04–0.24) for household income, 0.11 (0.01–0.21) for health insurance subsidy, 0.17 (0.04–0.30) for struggling to make ends meet, and 0.16 (0.01–0.31) for financial hardship (Table 2).

In the models including multimorbidity as the mediator, the risk of developing frailty was 1.57 (1.12–2.02) for those with lower educational level, 1.61 (1.10–2.11) for those with lower occupational positions, 2.56 (1.83–3.28) for those with lower household income, 2.18 (1.62–2.74) for those receiving health insurance subsidy, 2.59 (1.97–3.22) for those struggling to make ends meet, and 2.47 (1.75–3.18) for individuals having experienced financial hardship. Of these associations, the overall proportion due to mediation by multimorbidity was 0.39 (0.16–0.61) for educational level, 0.30 (0.10–0.50) for occupational position, 0.24 (0.15–0.34) for household income, 0.21 (0.09–0.33) for health insurance subsidy, 0.21 (0.11–0.31) for struggling to make ends meet, and 0.23 (0.09–0.38) for financial hardship (Table 2).

**Supplementary table 4** shows the result estimates of the four-way decomposition mediation analysis further adjusted for smoking, BMI and feeling of isolation; the same results pattern was observed, albeit with attenuated estimates. **Supplementary tables 5** and **6** display the results of the mediation analyses when considering pre-frailty as the outcome. Overall, the mediation role of chronic conditions on the association between socioeconomic disadvantage and subsequent pre-frailty was smaller than those observed on frailty.

In sensitivity analyses, repeating the mediation analyses excluding participants with baseline BMI<18.5 or >40 kg/m^2^, and then excluding participants with baseline mini nutritional assessment score <24 (i.e., at risk of malnutrition or in malnutrition) yielded predominantly similar results (**Supplementary tables 7** and **8**).

## Discussion

In this study of older adults in Lausanne, Switzerland, we found that chronic conditions, namely obesity and diabetes, mediated a substantial portion of the association between socioeconomic disadvantage and frailty. Participants with baseline socioeconomic disadvantage had a 1.5- to 2.5-fold higher risk of developing frailty compared with their socioeconomically advantaged counterparts. The proportion of the increased frailty risk explained by obesity, diabetes, and multimorbidity ranged from 11% to 55%.

Our results consistently showed that socioeconomic disadvantage was associated with a higher burden of chronic conditions, in line with extensive prior literature [8,9,21]. In line with previous research, socioeconomic disadvantage was also associated with increased frailty risk [7,22], and chronic conditions were associated with higher frailty and pre-frailty risk [[4], [5], [6]].

Few studies have explored the mediating role of chronic conditions on the longitudinal association between socioeconomic disadvantage and frailty [7]. A Dutch study among adults aged ≥65 years found that chronic disease multimorbidity explained 20% of the association between lower educational level and frailty [23]. Similarly, a Spanish study found that obesity and multimorbidity mediated 23% and 12%, respectively, of the association between lower educational level and frailty [24]. Another study of British civil servants showed that overweight and obesity mediated 11% of the association between lower occupational position and frailty, with minimal mediation by hypertension, diabetes, and cardiovascular disease [25].

Our study revealed larger mediation proportions, which may be attributed to differences in sample size, demographics, health profiles, and follow-up durations. For instance, the Dutch study had a higher baseline frailty prevalence (10.8% vs. 1.8% in our study) [23] and a higher attrition rate over follow-up (74% vs. 50% in our study). Follow-up in the Spanish study was limited to 3.5 years [24]. Our larger analytical sample and longer follow-up likely provided greater statistical power and more opportunities to detect mediation. While the Dutch study used a similar categorization of educational level to ours, the Spanish study classified occupational position as manual versus non-manual, and the British study used civil service occupation grades [25]. Importantly, while all previous studies based their mediation analyses on the "difference method," our use of the four-way decomposition counterfactual mediation method likely enabled us to capture a greater extend of the mediating role of chronic conditions [18].

### Implications for public health

Our finding that obesity and diabetes mediate a substantial proportion of the association between socioeconomic disadvantage and frailty has important implications for public health and clinical practice. It underscores the central role of primary care in reducing socioeconomic inequities in frailty through early screening, timely diagnosis, and effective management of chronic conditions, particularly obesity and diabetes.

In Switzerland, socioeconomically disadvantaged individuals have a markedly higher prevalence of diabetes [26], with a substantial proportion remaining undiagnosed [27], highlighting the need to prioritize screening and early treatment among vulnerable populations. Addressing social determinants of health—such as health literacy, food insecurity, mobility, language barriers, and financial hardship—is essential to ensure equitable care delivery [28].

Multidisciplinary care models involving primary care physicians, nurses, endocrinologists, social workers, and community health workers have been shown to improve diabetes outcomes in socioeconomically disadvantaged groups [28,29]. Improved glycemic control and weight loss are in turn associated with a lower risk of frailty [30,31].

However, effective chronic disease management also depends on broader structural conditions. Addressing social determinants of health requires systemic interventions to reduce inequalities in access to effective prevention and care. Timely, high-quality management of chronic conditions is strongly shaped by socioeconomic conditions through mechanisms such as health insurance coverage, out-of-pocket costs, and differential availability of services and medications [[32], [33], [34]]. These structural barriers may disproportionately restrict access to effective care among socioeconomically disadvantaged individuals, despite higher levels of need, thereby reinforcing socioeconomic gradients in chronic disease burden and, ultimately, frailty risk [[35], [36], [37], [38], [39]]. Addressing such inequities in healthcare access is therefore an important complement to broader public health strategies aimed at preventing obesity, diabetes, and their downstream consequences.

Given the strong links between obesity, diabetes, diet, and physical activity, population-level interventions to improve diet quality and promote physical activity are essential for reducing socioeconomic inequalities in chronic conditions and frailty [40,41]. Although knowledge-based public health campaigns play an important role, their effectiveness depends on concurrent structural changes to the food and built environments; without such changes, they may inadvertently widen inequalities by disproportionately benefiting socioeconomically advantaged groups [28,40,42]. Structural interventions include subsidies for healthy foods, regulation of food reformulation and advertising, taxation of unhealthy products [40], and investments in safe, accessible infrastructure for physical activity, such as recreational spaces and bicycle lanes [43].

Nevertheless, persistent socioeconomic inequalities in diet [44], physical activity [45], obesity, and diabetes [26] highlight the need for targeted interventions addressing barriers faced specifically by disadvantaged populations [29,46]. These include community-driven health programs [47], free or low-cost health screenings [48], and culturally tailored health promotion initiatives [29,46].

### Strengths and limitations

Strengths of this study include the use of six socioeconomic indicators capturing multiple dimensions of disadvantage; long follow-up (up to 15 years) enabling survival analyses; high participant retention compared with similar cohorts of older adults [15], reducing selection and attrition bias, and increasing statistical power, internal validity and reliability of our findings; and the use of four-way counterfactual mediation to quantify both total and mediated associations between socioeconomic disadvantage and frailty.

Several limitations should be considered. Chronic conditions and some frailty components were self-reported, and measures of disease management were unavailable (e.g., HbA1c levels, blood pressure), potentially underestimating disease burden. Excluded participants were more socioeconomically disadvantaged and had higher frailty prevalence, likely leading to underestimation of socioeconomic inequalities and mediation effects. We did not construct a composite socioeconomic status index; although multiple socioeconomic dimensions were evaluated, a composite measure may have captured broader socioeconomic gradients. Use of baseline rather than time-updated chronic conditions likely underestimated their mediating role [49], particularly for cardiovascular and respiratory diseases, which were relatively infrequent at ages 65–70. Given the observational design of the study, causal inference is limited. Finally, death could not be modeled as a competing risk because mortality data were unavailable and counterfactual mediation methods do not currently accommodate competing risks [18]; however, as competing-risk bias mainly affects cumulative incidence and mortality before frailty was low, the impact on hazard ratio estimates is likely minimal [50,51]. Future studies should incorporate biomarkers of disease control, improve retention of socioeconomically disadvantaged participants, and use time-dependent measures of exposures, mediators, and outcomes.

## Conclusion

Our findings suggest that chronic conditions, particularly obesity and diabetes, play a key role in mediating the long-term association between socioeconomic disadvantage and frailty. Targeted early screening, diagnosis, and management of these conditions among socioeconomically disadvantaged individuals, along with structural interventions to prevent obesity and diabetes, could help reduce socioeconomic inequities in frailty.

## Ethical statement

The Lc65+ study received approval from the Cantonal Human Research Ethical Committee, Vaud, Switzerland (Protocol 19/04). Participants were informed of the study goals and design and provided written informed consent.

## Data and code availability

The data are available to use upon submission of data request form and approval by the research committee. The analytical code is available upon request from first author.

## Declaration of Generative AI and AI-assisted technologies in the writing process

No Generative AI or AI-assisted technologies were used in the writing process.

## CRediT authorship contribution statement

**Carlos de Mestral:** Writing – review & editing, Writing – original draft, Visualization, Methodology, Formal analysis, Data curation, Conceptualization. **Saman Khalatbari-Soltani:** Writing – review & editing, Writing – original draft, Visualization, Methodology, Formal analysis, Conceptualization. **Patrick Bodenmann:** Writing – review & editing, Supervision, Conceptualization. **Yves Henchoz:** Writing – review & editing, Validation, Supervision, Resources, Project administration, Investigation, Funding acquisition, Data curation, Conceptualization. **Mauricio Avendano:** Writing – review & editing, Supervision, Resources, Project administration, Methodology, Funding acquisition, Formal analysis, Data curation, Conceptualization.

## Declaration of competing interest

The authors declare no conflict of interest.

## Appendix Supplementary materials

Image, application 1

Image, application 2

## References

[1] Hoogendijk E.O., Afilalo J., Ensrud K.E, Kowal P., Onder G., Fried L.P. (2019 Oct 12). Frailty: implications for clinical practice and public health. Lancet.

[2] Fried L.P, Tangen C.M, Walston J., Newman A.B, Hirsch C., Gottdiener J. (2001 Mar 1). Frailty in older adults: evidence for a phenotype. J Gerontol: A.

[3] Clegg A., Young J., Iliffe S., Rikkert M.O, Rockwood K. (2013 Mar 2). Frailty in elderly people. Lancet.

[4] Yuan L., Chang M., Wang J. (2021 July 1). Abdominal obesity, body mass index and the risk of frailty in community-dwelling older adults: a systematic review and meta-analysis. Age Ageing.

[5] Assar M.E, Laosa O., Rodríguez Mañas L. (2019). Diabetes and frailty. Curr Opin Clin Nutr Metab Care.

[6] Aprahamian I., Sassaki E., dos Santos M.F, Izbicki R., Pulgrossi R.C, Biella M.M (2018). Hypertension and frailty in older adults. J Clin Hypertens.

[7] Wang J., Hulme C. (2021 Apr 30). Frailty and socioeconomic status: a systematic review. J Public Health Res.

[8] Wagg E., Blyth F.M, Cumming R.G, Khalatbari-Soltani S. (2021 Aug 1). Socioeconomic position and healthy ageing: a systematic review of cross-sectional and longitudinal studies. Ageing Res Rev.

[9] Stringhini S., Carmeli C., Jokela M., Avendaño M., Muennig P., Guida F. (2017 Mar 25). Socioeconomic status and the 25 × 25 risk factors as determinants of premature mortality: a multicohort study and meta-analysis of 1·7 million men and women. Lancet.

[10] Yusuf S., Joseph P., Rangarajan S., Islam S., Mente A., Hystad P. (2020 Mar 7). Modifiable risk factors, cardiovascular disease, and mortality in 155 722 individuals from 21 high-income, middle-income, and low-income countries (PURE): a prospective cohort study. Lancet.

[11] Bauer U.E, Briss P.A, Goodman R.A, Bowman B.A. (2014 July 5). Prevention of chronic disease in the 21st century: elimination of the leading preventable causes of premature death and disability in the USA. Lancet.

[12] Hoffmann R., Kröger H., Pakpahan E. (2018 June 1). Pathways between socioeconomic status and health: does health selection or social causation dominate in Europe?. Adv Life Course Res.

[13] Seabrook J.A, Avison W.R. (2012). Socioeconomic status and cumulative disadvantage processes across the life course: implications for health outcomes. Can Rev Sociol/Rev Can Sociol.

[14] Swiss Federal Statistical Office. Lausanne - statistics [Internet]. [cited 2024 June 20]. Available from: https://www.bfs.admin.ch/bfs/en/home/statistiken/querschnittsthemen/city-statistics/staedteportraets/lausanne.html.

[15] Henchoz Y., Blanco J.M, Fustinoni S., Nanchen D., Büla C., Seematter-Bagnoud L. (2021 Nov 29). Cohort profile: the Lausanne cohort 65+ (Lc65+). Int J Epidemiol.

[16] Vellas B., Villars H., Abellan G., Soto M.E, Rolland Y., Guigoz Y. (2006). Overview of the MNA–its history and challenges. J Nutr Health Aging.

[17] Santos-Eggimann B., Karmaniola A., Seematter-Bagnoud L., Spagnoli J., Büla C., Cornuz J. (2008 Aug 18). The Lausanne cohort Lc65+: a population-based prospective study of the manifestations, determinants and outcomes of frailty. BMC Geriatr.

[18] VanderWeele T.J. (2014). A unification of mediation and interaction: a 4-way decomposition. Epidemiology.

[19] van Zwieten A., Tennant P.W.G., Kelly-Irving M., Blyth F.M, Teixeira-Pinto A., Khalatbari-Soltani S. (2022). Avoiding overadjustment bias in social epidemiology through appropriate covariate selection: a primer. J Clin Epidemiol.

[20] Discacciati A., Bellavia A., Lee J.J, Mazumdar M., Valeri L. (2018 Nov 16). Med4way: a Stata command to investigate mediating and interactive mechanisms using the four-way effect decomposition. Int J Epidemiol.

[21] de Mestral C., Stringhini S. (2017). Socioeconomic status and cardiovascular disease: an update. Curr Cardiol Rep.

[22] Dugravot A., Fayosse A., Dumurgier J., Bouillon K., Rayana T.B, Schnitzler A. (2020 Jan 1). Social inequalities in multimorbidity, frailty, disability, and transitions to mortality: a 24-year follow-up of the Whitehall II cohort study. Lancet Public Health.

[23] Hoogendijk E.O, van Hout H.P.J., Heymans M.W, van der Horst H.E, Frijters D.H.M., Broese van Groenou M.I (2014). Explaining the association between educational level and frailty in older adults: results from a 13-year longitudinal study in the Netherlands. Ann Epidemiol.

[24] Soler-Vila H., García-Esquinas E., León-Muñoz L.M, López-García E., Banegas J.R, Rodríguez-Artalejo F. (2016 Apr 1). Contribution of health behaviours and clinical factors to socioeconomic differences in frailty among older adults. J Epidemiol Community Health.

[25] Brunner E.J, Shipley M.J, Ahmadi-Abhari S., Valencia Hernandez C., Abell J.G, Singh-Manoux A. (2018). Midlife contributors to socioeconomic differences in frailty during later life: a prospective cohort study. Lancet Public Health.

[26] de Mestral C., Piumatti G., Nehme M., Guessous I., Stringhini S. (2024). Twelve–year (2008–2019) trends in socioeconomic inequalities in cardiovascular risk factors in a Swiss representative survey of the general population. Prev Med Rep.

[27] de M.C, S S., Guessous I., Jornayvaz F.R (2020 July 1). Thirteen-year trends in the prevalence of diabetes according to socioeconomic condition and cardiovascular risk factors in a Swiss population. BMJ Open Diabetes Res Care.

[28] Hill-Briggs F., Adler N.E, Berkowitz S.A, Chin M.H, Gary-Webb T.L, Navas-Acien A. (2021). Social determinants of health and diabetes: a scientific review. Diabetes Care.

[29] Gkiouleka A., Wong G., Sowden S., Bambra C., Siersbaek R., Manji S. (2023 June 1). Reducing health inequalities through general practice. Lancet Public Health.

[30] Simpson F.R, Justice J.N, Pilla S.J, Kritchevsky S.B, Boyko E.J, Munshi M.N (2022 Dec 21). An examination of whether diabetes control and treatments are associated with change in frailty index across 8 years: an ancillary exploratory study from the action for health in diabetes (look AHEAD) trial. Diabetes Care.

[31] Hazuda H.P, Pan Q., Florez H., Luchsinger J.A, Crandall J.P, Venditti E.M (2021 Apr 30). Association of intensive lifestyle and metformin interventions with frailty in the diabetes prevention program outcomes study. J Gerontol A Biol Sci Med Sci.

[32] Hart J.T. (1971 Feb 27). The inverse care law. Lancet.

[33] Cesare M.D, Khang Y.H, Asaria P., Blakely T., Cowan M.J, Farzadfar F. (2013). Inequalities in non-communicable diseases and effective responses. Lancet.

[34] Shi L., Chen C.C, Nie X., Zhu J., Hu R. (2014). Racial and socioeconomic disparities in access to primary care among people with chronic conditions. J Am Board Fam Med.

[35] Karagiannis T., Bekiari E., Tsapas A. (2023 Oct 1). Socioeconomic aspects of incretin-based therapy. Diabetologia.

[36] Eberly L.A, Yang L., Essien U.R, Eneanya N.D, Julien H.M, Luo J. (2021 Dec 17). Racial, ethnic, and socioeconomic inequities in glucagon-like peptide-1 receptor agonist use among patients with diabetes in the US. JAMA Health Forum.

[37] Falkentoft A.C, Andersen J., Malik M.E, Selmer C., Gæde P.H, Staehr P.B (2022 Mar 1). Impact of socioeconomic position on initiation of SGLT-2 inhibitors or GLP-1 receptor agonists in patients with type 2 diabetes – a Danish nationwide observational study. Lancet Reg Health – Eur [Internet].

[38] Lyu B., Chang A.R, Inker L.A, Selvin E., Grams M.E, Shin J.I. (2022 Apr 2). Socioeconomic status and use of obesogenic and anti-obesity medications in the United States: a population-based study. Lancet Reg Health Am.

[39] Pazzagli L., Trolle Lagerros Y. (2024 Nov 26). Socioeconomic and demographic inequalities in off-label prescription of glucagon-like peptide-1 receptor agonists: a Swedish descriptive cohort study. Obes Facts.

[40] Mozaffarian D. (2016 Jan 12). Dietary and policy priorities for cardiovascular disease, diabetes, and obesity. Circulation.

[41] Zhang Y., Pan X.F, Chen J., Xia L., Cao A., Zhang Y. (2020 Jan 1). Combined lifestyle factors and risk of incident type 2 diabetes and prognosis among individuals with type 2 diabetes: a systematic review and meta-analysis of prospective cohort studies. Diabetologia.

[42] McGill R., Anwar E., Orton L., Bromley H., Lloyd-Williams F., O’Flaherty M. (2015 May 2). Are interventions to promote healthy eating equally effective for all? Systematic review of socioeconomic inequalities in impact. BMC Public Health.

[43] Hernández E.D, Cobo E.A, Cahalin L.P, Seron P. (2023). Impact of structural-level environmental interventions on physical activity: a systematic review. Int Arch Occup Environ Health.

[44] de Mestral C. (2018).

[45] Ball K., Carver A., Downing K., Jackson M., O’Rourke K. (2015). Addressing the social determinants of inequities in physical activity and sedentary behaviours. Health Promot Int.

[46] Ruddock J.S, Poindexter M., Gary-Webb T.L, Walker E.A, Davis N.J (2016). Innovative strategies to improve diabetes outcomes in disadvantaged populations. Diabet Med.

[47] O’Mara-Eves A., Brunton G., Oliver S., Kavanagh J., Jamal F., Thomas J. (2015 Feb 12). The effectiveness of community engagement in public health interventions for disadvantaged groups: a meta-analysis. BMC Public Health.

[48] Gharacheh L., Amini-Rarani M., Torabipour A., Karimi S. (2024). A scoping review of possible solutions for decreasing socioeconomic inequalities in type 2 diabetes mellitus. Int J Prev Med.

[49] Groeniger J.O, van Lenthe F.J (2016 Oct 1). Contribution of time-varying measures of health behaviours to socioeconomic inequalities in mortality: how to understand the underlying mechanisms?. J Epidemiol Community Health.

[50] Austin P.C, Fine J.P. (2017 Nov 30). Practical recommendations for reporting fine-gray model analyses for competing risk data. Stat Med.

[51] Hernán M.A. (2010). The hazards of hazard ratios. Epidemiology.

