# Supplementary material for: The mediating role of chronic disease in socioeconomic inequalities in frailty: A longitudinal cohort study of older adults in Lausanne, Switzerland
Source: J Frailty Aging. 2026 Mar 20;15(3):100134. doi: 10.1016/j.tjfa.2026.100134 (PMC13019564; doi:10.1016/j.tjfa.2026.100134)
Supplement: Supplementary file 2 [file mmc2.docx]

**Supplementary material**

**The mediating role of chronic disease in socioeconomic inequalities in frailty: a longitudinal cohort study of older adults in Lausanne, Switzerland**

Carlos de Mestral, Saman Khalatbari-Soltani, Patrick Bodenmann, Yves Henchoz, Mauricio Avendano

**Supplementary table 1.** Assessment of socioeconomic disadvantage, chronic conditions, and of frailty according to the Fried phenotype

| **Socioeconomic indicator** | **Assessment method** |
| --- | --- |
| Educational level | Participants reported their highest completed educational level, which we grouped using the International Standard Classification of Education (ISCED)^32^ as lower (basic compulsory education; ISCED levels 0-2), middle (apprenticeship; ISCED level 3), and higher (post compulsory; ISCED levels 4-8). |
| Occupational position | Participants also reported their current (or last) occupational position, which we categorized as lower (worker/farmer, unqualified employee, unqualified laborer), middle (qualified employee), or higher (high managerial, middle managerial, or independent). |
| Household income | Participants were asked to report their household income by indicating their gross monthly household income on a scale (ranging from 0 to ≥12'500 CHF), from which household income was divided into tertiles for all cohorts combined as lower (<4270 CHF), middle (4207-6650 CHF), high (≥6651) (1 1 CHF ≈ 0.93 GBP as of October 13, 2025). |

| **Chronic conditions** | **Assessment method** |
| --- | --- |
| Obesity, hypertension, diabetes, cardiovascular disease, chronic respiratory disease | Participants were asked "Has a doctor told you that you have...? (many conditions possible)" and "Do you currently take at least once per week any of the following medications? (many medications possible)"  Obesity was additionally assessed during physical examination via BMI calculation from weight and height measurements. |

| **Frailty score component** | **Assessment method** |
| --- | --- |
| Unintentional weight loss | Self-reported unintentional weight loss > 5 kg in prior year |
| Exhaustion | Self-reported exhaustion to question : *Did you have feelings of generalized*  *weakness, weariness, or lack of energy in the last 4 weeks?* |
| Low physical activity | Self-reported <20 minutes of sports activity once a week, <30 minutes total of walking per day three times per week and avoiding stairs climbing or light weight carrying in daily activities. |
| Muscular weakness | Low grip strength, defined as lowest 20% of maximal grip strength in the dominant hand (3 measured averaged) using a hand-held dynamometer, adjusted for gender and BMI. |
| Slow walking speed | Low gait speed in 20-meter walk test, defined as slowest 20% according to gender and height. |

Reference: Fried LP, Tangen CM, Walston J, et al. Frailty in Older Adults: Evidence for a Phenotype. *The Journals of Gerontology: Series A* 2001; 56: M146–57.; Santos-Eggimann B, Karmaniola A, Seematter-Bagnoud L, et al. The Lausanne cohort Lc65+: a population-based prospective study of the manifestations, determinants and outcomes of frailty. *BMC Geriatr* 2008; 8: 20.

**Supplementary figure 1.** Flowchart of participant selection

Participants in Lc65+ with baseline assessment

N = 4 731

**Main analytical sample**

Participants with complete socioeconomic, demographic, chronic condition, and frailty data

N = 3 643

Excluded participants with missing socioeconomic, demographic, chronic condition, and frailty data

N = 1 088

**Sensitivity analysis 1**

Excluding participants with BMI <18.5 or >40

N = 3 537

**Sensitivity analysis 2**

Excluding participants with Mini Nutritional Assessment score <24

N = 3 044

Flowchart of participant selection for the main analytical sample and sensitivity analyses. Participants excluded from the main analysis were removed due to missing socioeconomic, demographic, chronic condition, or frailty data. Sensitivity analyses were performed in subsamples derived from the main analytical sample by excluding individuals with BMI <18.5 or >40, and those with a Mini Nutritional Assessment score <24.

**Supplementary table 2**. Baseline characteristics of analytical sample with excluded participants

|  | **Included** | **Excluded** | ***p-value*** |
| --- | --- | --- | --- |
| **N** | 3643 | 1088 |  |
| **Age** (median, IQR), y | 68 (66-70) | 68 (66-70) |  |
| **Men** | 1507 (41.4) | 488 (44.9) | *0.04* |
| **Women** | 2136 (58.6) | 600 (55.1) |  |
| **Educational level** |  |  | *<0.01* |
| Basic compulsory | 637 (17.6) | 198 (30.7) |  |
| Apprenticeship | 1422 (39.3) | 240 (37.2) |  |
| Post compulsory schooling | 1558 (43.1) | 208 (32.2) |  |
| **Occupational position** |  |  | *<0.01* |
| Lower | 681 (19.2) | 195 (32.6) |  |
| Middle | 1466 (41.2) | 214 (35.8) |  |
| Higher | 1407 (39.6) | 189 (31.6) |  |
| **Household income** |  |  | *<0.01* |
| Lowest tertile | 1194 (33.5) | 103 (49.0) |  |
| Middle tertile | 1171 (32.9) | 64 (30.5) |  |
| Higher tertile | 1194 (33.5) | 43 (20.5) |  |
| **Receives partial/full insurance subsidy** | 511 (14.0) | 272 (25.8) | *<0.01* |
| **Struggles making ends meet** | 506 (13.9) | 77 (21.1) | *<0.01* |
| **Financial hardship in past year** | 281 (7.7) | 77 (11.2) | *<0.01* |
| **Isolation feeling** |  |  | *0.06* |
| Rarely/never | 2774 (76.1) | 168 (70.0) |  |
| Sometimes | 634 (17.4) | 49 (20.4) |  |
| Frequently/always | 235 (6.5) | 23 (9.6) |  |
| **Current smoker** | 625 (17.2) | 279 (26.5) | *<0.01* |
| **Frailty score** |  |  | *<0.01* |
| Non-frailty | 2518 (71.6) | 175 (53.8) |  |
| Pre-frailty | 933 (26.6) | 126 (38.8) |  |
| Frailty | 63 (1.8) | 24 (7.4) |  |
| **Obesity** | 833 (22.9) | 95 (27.0) | *0.08* |
| **Diabetes** | 314 (8.6) | 92 (14.7) | *<0.01* |
| **Hypertension** | 1245 (34.2) | 215 (34.4) | *0.91* |
| **Cardiovascular disease** | 376 (10.6) | 69 (18.6) | *<0.01* |
| **Chronic respiratory disease** | 300 (8.2) | 59 (9.4) | *0.32* |
| **Multimorbidity** | 803 (22.0) | 135 (20.4) | *0.34* |

**Supplementary figure 2**. Association between socioeconomic condition and chronic conditions at baseline, Lausanne Cohort 65+


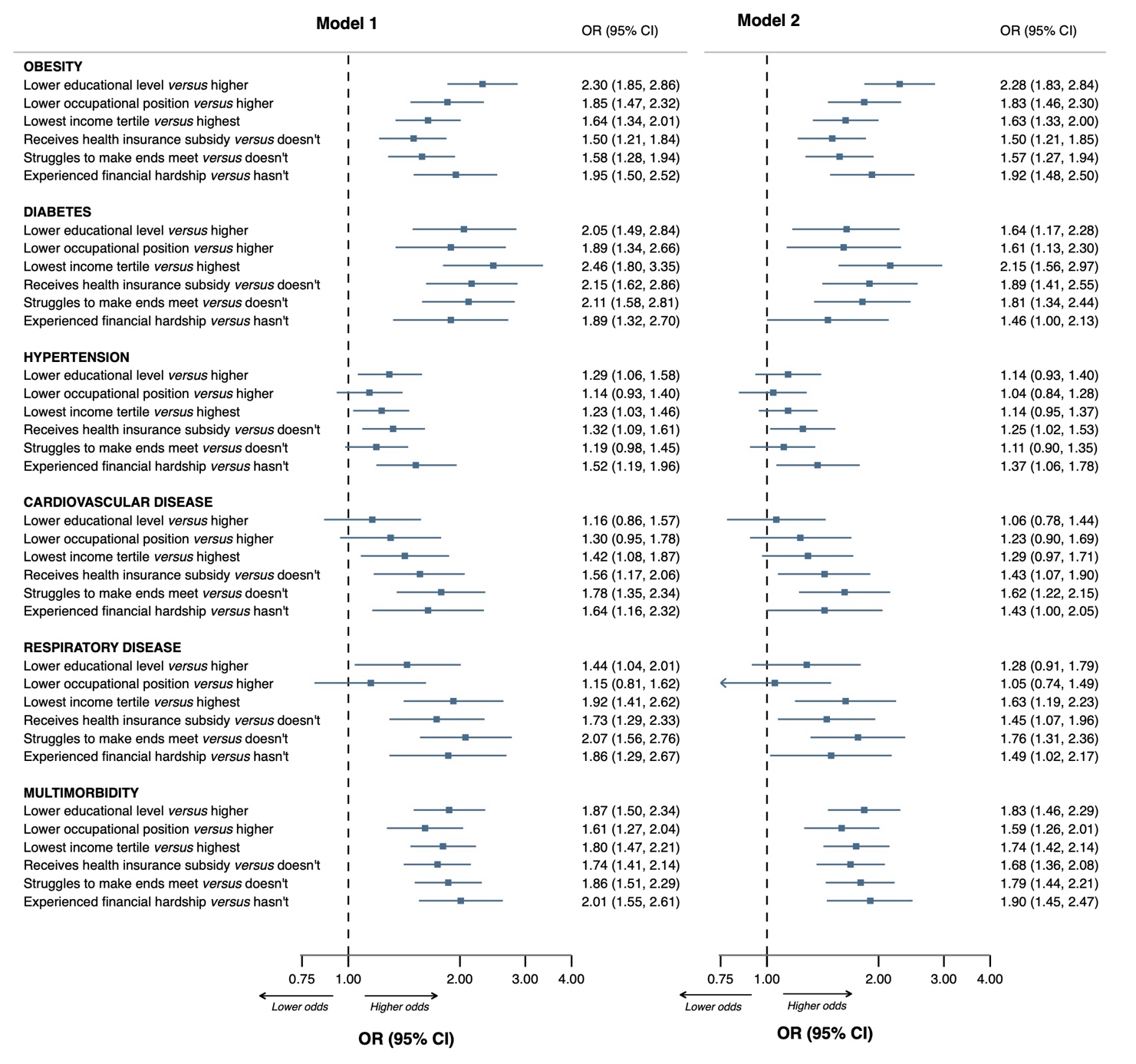


Results are expressed as Odds ratio (95% confidence interval), adjusted for age, sex, and cohort in Model 1, and additionally for smoking and BMI (except obesity model) in Model 2, from Logistic regression. Multimorbidity indicates the presence of at least 2 chronic conditions. * Relative to participants who answered "no." Sample size = 3643.

**Supplementary figure 3.** Association between socioeconomic condition at baseline and subsequent frailty and pre-frailty, Lausanne cohort 65+


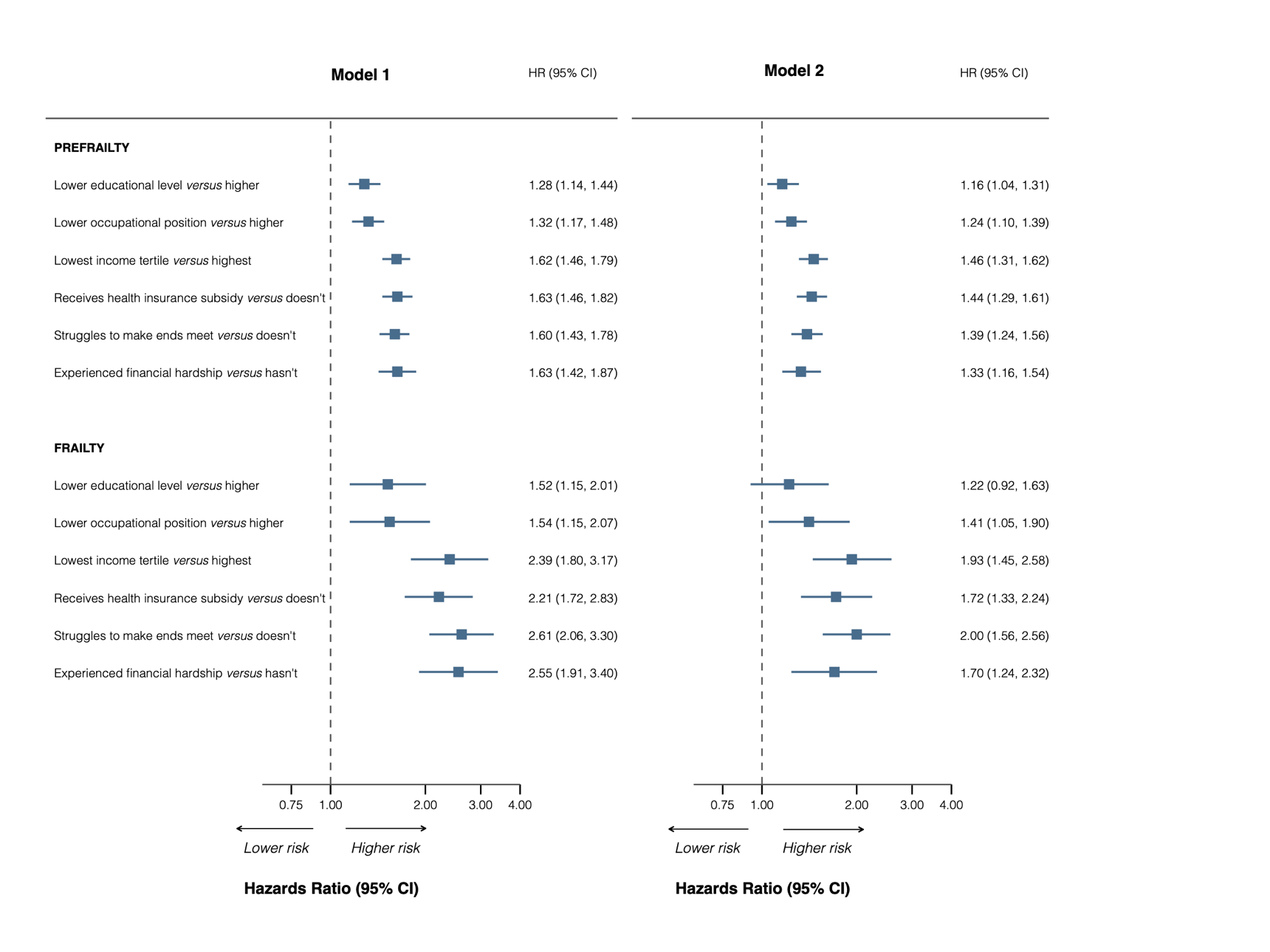


Results are Hazards ratios (95% confidence interval), adjusted for age, sex, and cohort in Model 1, and additionally for smoking, feeling of isolation, and BMI in Model 2, from Cox proportional hazards regression. Sample size = 3643.

**Supplementary table 3**. Longitudinal association between socioeconomic disadvantage and subsequent frailty, with chronic conditions treated as potential confounders

| Exposure | Model 1 | Model 1  + obesity | Model  + diabetes | Model 1  + hypertension | Model 1  + cardiovascular disease | Model 1  + chronic respiratory disease | Model 1  + multimorbidity |
| --- | --- | --- | --- | --- | --- | --- | --- |
|  | HR (95% CI) | HR (95% CI) | HR (95% CI) | HR (95% CI) | HR (95% CI) | HR (95% CI) | HR (95% CI) |
| Educational level | 1.52 (1.15-2.01) | 1.38 (1.06-1.79) | 1.47 (1.13-1.95) | 1.50 (1.12-1.98) | 1.42 (1.14-2.02) | 1.46 (1.10-1.94) | 1.46 (1.13-1.90) |
| Occupational position | 1.54 (1.15-2.07) | 1.48 (1.13-1.95) | 1.50 (1.11-2.02) | 1.49 (1.10-2.01) | 1.56 (1.16-2.09) | 1.54 (1.15-2.06) | 1.54 (1.17-2.03) |
| Household income | 2.39 (1.80-3.17) | 2.29 (1.75-3.02) | 2.30 (1.76-3.02) | 2.34 (1.36-3.11) | 2.40 (1.80-3.19) | 2.38 (1.82-3.13) | 2.26 (1.73-2.96) |
| Health insurance subsidy | 2.21 (1.72-2.83) | 2.12 (1.67-2.69) | 2.14 (1.68-2.72) | 2.16 (1.68-2.77) | 2.25 (1.75-2.88) | 2.21 (1.74-2.81) | 2.06 (1.62-2.63) |
| Struggle to make ends meet | 2.61 (2.06-3.30) | 2.43 (1.93-3.06) | 2.54 (2.02-3.19) | 2.58 (2.04-3.27) | 2.60 (2.05-3.31) | 2.58 (2.05-3.25) | 2.42 (1.92-3.04) |
| Financial hardship | 2.55 (1.91-3.40) | 2.24 (1.70-2.97) | 2.47 (1.88-3.26) | 2.46 (1.84-3.29) | 2.67 (2.02-3.54) | 2.51 (1.90-3.34) | 2.27 (1.72-3.01) |

Results are Hazards ratio (95% confidence interval) from Cox proportional hazards regression, with estimate adjusted for age, sex, and cohort in Model 1, and additionally for each chronic condition, respectively.

**Supplementary figure 4**. Association between chronic condition at baseline and subsequent prefrailty and frailty, Lausanne cohort 65+ cohort


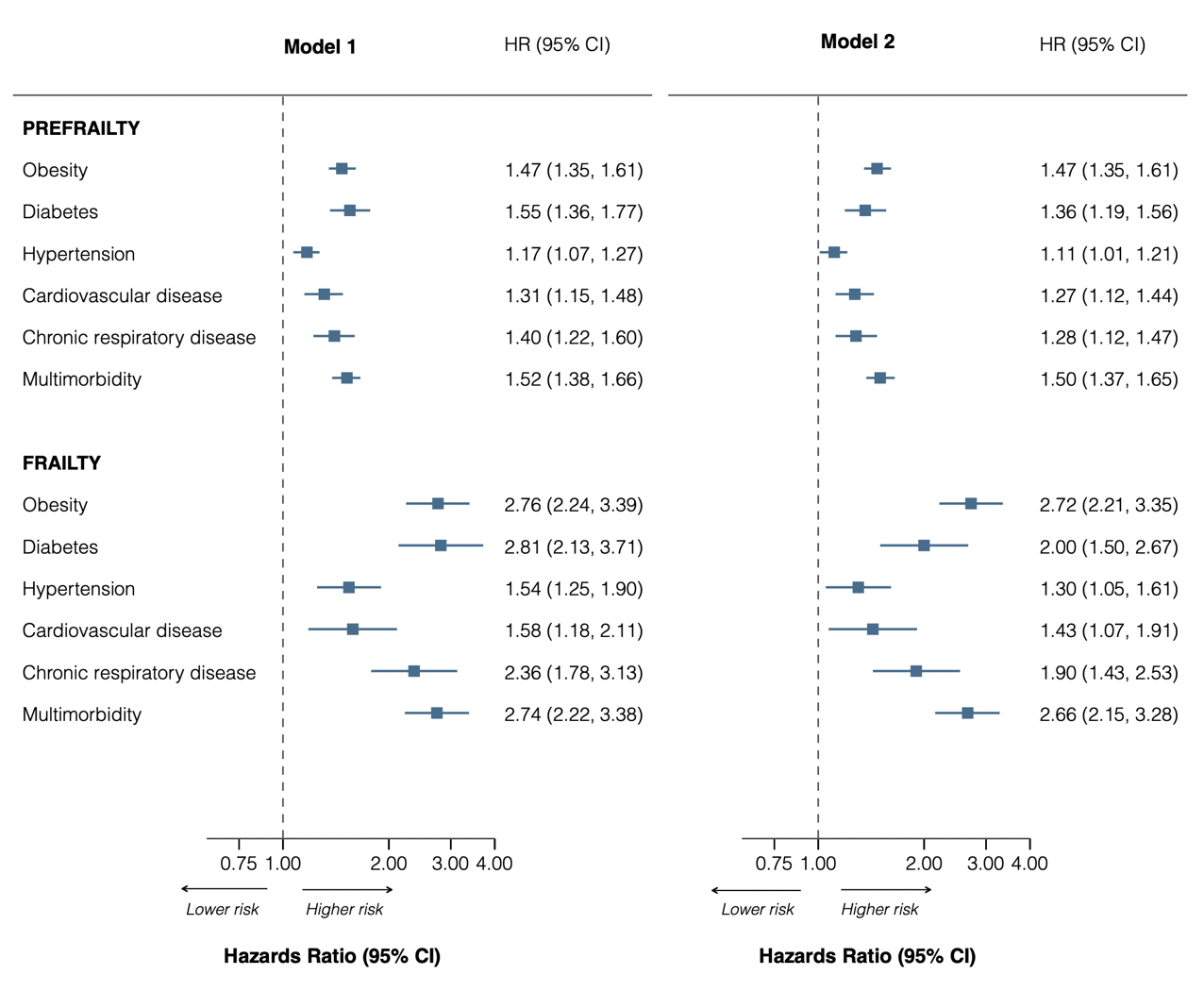


Results are Hazards ratios (95% confidence interval), adjusted for age, sex, and cohort in Model 1, and additionally for smoking, feeling of isolation, and BMI (except model with obesity as exposure), from Cox proportional hazards regression. Sample size = 3643

**Supplementary table 4**. Mediation of chronic disease in the association between socioeconomic condition and subsequent frailty, Lausanne Cohort 65+

| **Exposure** | **Mediator** | **Total risk**  **HR (95% CI)** | **Proportion of direct association**  **(95% CI)** | **Overall proportion due to mediation**  **(95% CI)** | **Overall proportion due to interaction**  **(95% CI)** | **Overall portion eliminated**  **(95% CI)** |
| --- | --- | --- | --- | --- | --- | --- |
| Obesity | Educational level | 1.46 (1.05-1.88) | 0.19 (-0.40-0.78) | 0.58 (0.19-0.98) | 0.43 (-0.19-1.04) | 0.81 (0.22-1.40) |
|  | Occupational position | 1.59 (1.10-2.08) | 0.36 (-0.11-0.83) | 0.37 (0.13-0.61) | 0.44 (-0.06-0.93) | 0.64 (0.17-1.11) |
|  | Household income | 2.16 (1.55-2.78) | 0.53 (0.29-0.78) | 0.20 (0.10-0.30) | 0.39 (0.13-0.65) | 0.47 (0.22-0.71) |
|  | Health insurance subsidy | 1.86 (1.39-2.33) | 0.61 (0.25-0.97) | 0.18 (0.05-0.31) | 0.28 (-0.09-0.64) | 0.39 (0.03-0.75) |
|  | Struggle to make ends meet | 2.11 (1.60-2.62) | 0.77 (0.47-1.08) | 0.13 (0.03-0.24) | 0.12 (-0.19-0.44) | 0.23 (-0.08-0.53) |
|  | Financial hardship | 1.93 (1.33-2.53) | 0.37 (-0.07-0.81) | 0.33 (0.13-0.53) | 0.47 (0.02-0.91) | 0.63 (0.19-1.07) |
| Diabetes | Educational level | 1.22 (0.87-1.57) | 0.92 (0.60-1.25) | 0.09 (-0.09-0.27) | -0.03 (-0.37-0.32) | 0.08 (-0.25-0.40) |
|  | Occupational position | 1.35 (0.94-1.76) | 0.88 (0.63-1.14) | 0.07 (-0.05-0.20) | 0.07 (-0.18-0.32) | 0.12 (-0.14-0.37) |
|  | Household income | 1.85 (1.32-2.38) | 0.92 (0.82-1.02) | 0.05 (0.00-0.11) | 0.06 (-0.05-0.17) | 0.08 (-0.02-0.18) |
|  | Health insurance subsidy | 1.73 (1.29-2.18) | 0.97 (0.83-1.10) | 0.04 (-0.03-0.11) | -0.01 (-0.14-0.12) | 0.03 (-0.10-0.17) |
|  | Struggle to make ends meet | 1.90 (1.43-2.37) | 0.90 (0.75-1.05) | 0.06 (-0.02-0.14) | 0.07 (-0.07-0.21) | 0.10 (-0.05-0.25) |
|  | Financial hardship | 1.62 (1.10-2.13) | 0.95 (0.77-1.12) | 0.04 (-0.04-0.11) | 0.02 (-0.15-0.19) | 0.05 (-0.12-0.23) |
| Hypertension | Educational level | 1.20 (0.87-1.54) | 1.68 (-0.13-3.50) | 0.01 (-0.07-0.10) | -0.76 (-2.71-1.18) | -0.68 (-2.50-1.13) |
|  | Occupational position | 1.38 (0.96-1.80) | 1.10 (0.34-1.86) | 0.01 (-0.03-0.05) | -0.11 (-0.88-0.66) | -0.10 (-0.86-0.66) |
|  | Household income | 1.93 (1.39-2.47) | 0.78 (0.46-1.09) | 0.02 (-0.01-0.06) | 0.22 (-0.11-0.54) | 0.22 (-0.09-0.54) |
|  | Health insurance subsidy | 1.70 (1.27-2.13) | 0.98 (0.50-1.46) | 0.02 (-0.04-0.08) | 0.00 (-0.49-0.48) | 0.02 (-0.46-0.50) |
|  | Struggle to make ends meet | 1.95 (1.49-2.42) | 0.93 (0.57-1.30) | 0.01 (-0.02-0.05) | 0.06 (-0.31-0.42) | 0.07 (-0.30-0.43) |
|  | Financial hardship | 1.67 (1.17-2.16) | 0.95 (0.31-1.60) | 0.04 (-0.08-0.15) | 0.01 (-0.64-0.66) | 0.05 (-0.60-0.69) |
| Cardiovascular disease | Educational level | 1.20 (0.86-1.54) | 1.29 (0.31-2.26) | 0.00 (-0.04-0.04) | -0.28 (-1.25-0.68) | -0.29 (-1.26-0.69) |
|  | Occupational position | 1.40 (0.97-1.83) | 0.99 (0.49-1.49) | 0.02 (-0.04-0.08) | -0.01 (-0.54-0.51) | 0.01 (-0.49-0.51) |
|  | Household income | 2.05 (1.45-2.66) | 0.77 (0.53-1.01) | 0.04 (-0.02-0.11) | 0.23 (-0.02-0.48) | 0.23 (-0.01-0.47) |
|  | Health insurance subsidy | 1.82 (1.33-2.31) | 0.82 (0.44-1.19) | 0.07 (-0.04-0.17) | 0.16 (-0.22-0.54) | 0.18 (-0.19-0.56) |
|  | Struggle to make ends meet | 2.03 (1.52-2.54) | 0.87 (0.57-1.17) | 0.06 (-0.04-0.16) | 0.10 (-0.21-0.41) | 0.13 (-0.17-0.43) |
|  | Financial hardship | 1.72 (1.17-2.27) | 0.96 (0.46-1.46) | 0.04 (-0.09-0.16) | 0.01 (-0.51-0.52) | 0.04 (-0.46-0.54) |
| Chronic respiratory disease | Educational level | 1.19 (0.85-1.52) | 0.89 (0.40-1.38) | 0.08 (-0.10-0.27) | 0.03 (-0.47-0.53) | 0.11 (-0.38-0.60) |
|  | Occupational position | 1.38 (0.96-1.79) | 0.91 (0.64-1.19) | 0.00 (-0.07-0.07) | 0.08 (-0.19-0.36) | 0.09 (-0.19-0.36) |
|  | Household income | 1.91 (1.37-2.45) | 0.91 (0.78-1.04) | 0.05 (-0.01-0.10) | 0.07 (-0.07-0.21) | 0.09 (-0.04-0.22) |
|  | Health insurance subsidy | 1.69 (1.26-2.12) | 0.92 (0.72-1.13) | 0.05 (-0.03-0.12) | 0.04 (-0.16-0.25) | 0.08 (-0.13-0.28) |
|  | Struggle to make ends meet | 1.96 (1.49-2.43) | 0.94 (0.77-1.11) | 0.05 (-0.03-0.12) | 0.02 (-0.16-0.19) | 0.06 (-0.11-0.23) |
|  | Financial hardship | 1.69 (1.19-2.20) | 1.02 (0.80-1.25) | 0.02 (-0.05-0.09) | -0.06 (-0.30-0.17) | -0.02 (-0.25-0.20) |
| Multimorbidity | Educational level | 1.47 (1.06-1.88) | 0.54 (0.03-1.04) | 0.32 (0.09-0.56) | 0.21 (-0.37-0.80) | 0.46 (-0.04-0.97) |
|  | Occupational position | 1.59 (1.11-2.07) | 0.52 (0.08-0.96) | 0.24 (0.07-0.42) | 0.33 (-0.15-0.80) | 0.48 (0.04-0.92) |
|  | Household income | 2.20 (1.57-2.82) | 0.38 (0.13-0.63) | 0.25 (0.14-0.36) | 0.57 (0.30-0.83) | 0.62 (0.37-0.87) |
|  | Health insurance subsidy | 1.91 (1.43-2.39) | 0.58 (0.23-0.93) | 0.21 (0.08-0.34) | 0.30 (-0.06-0.66) | 0.42 (0.07-0.77) |
|  | Struggle to make ends meet | 2.26 (1.72-2.80) | 0.58 (0.30-0.86) | 0.21 (0.10-0.32) | 0.31 (0.02-0.60) | 0.42 (0.14-0.70) |
|  | Financial hardship | 2.01 (1.42-2.60) | 0.60 (0.18-1.01) | 0.24 (0.07-0.42) | 0.25 (-0.18-0.68) | 0.40 (-0.01-0.82) |

Results are from counterfactual mediation models. Multimorbidity indicates having at least two chronic conditions (diabetes, hypertension, cardiovascular disease, respiratory disease). The total effect is Hazards ratio (95% CI) from Cox proportional hazards ratio, adjusted for baseline age, sex, cohort, smoking, BMI (except model with obesity as mediator), and isolation feeling. Proportion of direct association (95% CI) indicates the association between the exposure and the outcome that includes neither interaction nor mediation. Proportion due to mediation indicates the indirect association of the exposure with the outcome that involves pathways through the mediator. Proportion due to interaction indicates the association of the exposure with the outcomes via interaction with the mediator. Eliminated proportion indicates the proportion of the association that would be eliminated if the mediator were removed (e.g., fixed to 0). Sample size = 3643.

**Supplementary table 5**. Mediation of chronic disease in the association between socioeconomic condition and subsequent prefrailty, Lausanne Cohort 65+

| **Exposure** | **Mediator** | **Total risk**  **HR (95% CI)** | **Proportion of direct association**  **(95% CI)** | **Overall proportion due to mediation**  **(95% CI)** | **Overall proportion due to interaction**  **(95% CI)** | **Overall portion eliminated**  **(95% CI)** |
| --- | --- | --- | --- | --- | --- | --- |
| Obesity | Educational level | 1.24 (1.12-1.36) | 0.55 (0.22-0.89) | 0.41 (0.20-0.62) | 0.06 (-0.33-0.45) | 0.45 (0.11-0.78) |
|  | Occupational position | 1.24 (1.12-1.37) | 0.65 (0.32-0.97) | 0.30 (0.13-0.47) | 0.08 (-0.27-0.44) | 0.35 (0.03-0.68) |
|  | Household income | 1.49 (1.35-1.63) | 0.77 (0.61-0.93) | 0.14 (0.08-0.21) | 0.13 (-0.05-0.31) | 0.23 (0.07-0.39) |
|  | Health insurance subsidy | 1.49 (1.35-1.64) | 0.78 (0.57-0.98) | 0.12 (0.04-0.20) | 0.13 (-0.08-0.34) | 0.22 (0.02-0.43) |
|  | Struggle to make ends meet | 1.43 (1.29-1.57) | 0.89 (0.65-1.12) | 0.12 (0.04-0.21) | -0.01 (-0.26-0.23) | 0.11 (-0.12-0.35) |
|  | Financial hardship | 1.49 (1.32-1.66) | 0.66 (0.38-0.95) | 0.22 (0.09-0.35) | 0.18 (-0.10-0.46) | 0.34 (0.05-0.62) |
| Diabetes | Educational level | 1.23 (1.11-1.34) | 0.89 (0.72-1.07) | 0.11 (0.00-0.22) | -0.01 (-0.21-0.18) | 0.11 (-0.07-0.28) |
|  | Occupational position | 1.23 (1.10-1.35) | 0.95 (0.80-1.11) | 0.08 (-0.01-0.17) | -0.06 (-0.24-0.12) | 0.05 (-0.11-0.20) |
|  | Household income | 1.47 (1.33-1.60) | 0.96 (0.89-1.03) | 0.05 (0.01-0.10) | -0.03 (-0.12-0.07) | 0.04 (-0.03-0.11) |
|  | Health insurance subsidy | 1.48 (1.34-1.62) | 0.96 (0.84-1.08) | 0.06 (-0.01-0.13) | -0.03 (-0.16-0.09) | 0.04 (-0.08-0.16) |
|  | Struggle to make ends meet | 1.44 (1.31-1.58) | 0.94 (0.80-1.08) | 0.07 (-0.01-0.15) | -0.02 (-0.16-0.13) | 0.06 (-0.08-0.20) |
|  | Financial hardship | 1.47 (1.29-1.64) | 1.01 (0.86-1.17) | 0.03 (-0.04-0.10) | -0.08 (-0.25-0.09) | -0.01 (-0.17-0.14) |
| HTA | Educational level | 1.20 (1.08-1.33) | 1.24 (0.66-1.82) | 0.03 (-0.03-0.09) | -0.31 (-0.94-0.31) | -0.24 (-0.82-0.34) |
|  | Occupational position | 1.23 (1.10-1.36) | 0.99 (0.53-1.46) | 0.03 (-0.02-0.07) | -0.02 (-0.50-0.46) | 0.01 (-0.46-0.47) |
|  | Household income | 1.46 (1.33-1.60) | 0.94 (0.73-1.15) | 0.02 (0.00-0.05) | 0.04 (-0.18-0.27) | 0.06 (-0.15-0.27) |
|  | Health insurance subsidy | 1.48 (1.34-1.62) | 0.91 (0.63-1.18) | 0.03 (-0.01-0.07) | 0.07 (-0.21-0.35) | 0.09 (-0.18-0.37) |
|  | Struggle to make ends meet | 1.48 (1.33-1.62) | 0.70 (0.44-0.96) | 0.04 (-0.01-0.09) | 0.29 (0.03-0.55) | 0.30 (0.04-0.56) |
|  | Financial hardship | 1.47 (1.29-1.64) | 0.95 (0.58-1.33) | 0.04 (-0.04-0.11) | 0.01 (-0.37-0.39) | 0.05 (-0.33-0.42) |
| Cardiovascular disease | Educational level | 1.22 (1.09-1.35) | 0.99 (0.62-1.35) | 0.02 (-0.05-0.09) | 0.00 (-0.38-0.37) | 0.01 (-0.35-0.38) |
|  | Occupational position | 1.20 (1.07-1.33) | 1.19 (0.73-1.65) | 0.03 (-0.03-0.09) | -0.26 (-0.76-0.24) | -0.19 (-0.65-0.27) |
|  | Household income | 1.45 (1.31-1.59) | 0.97 (0.79-1.15) | 0.04 (0.00-0.09) | -0.01 (-0.22-0.19) | 0.03 (-0.15-0.21) |
|  | Health insurance subsidy | 1.47 (1.31-1.63) | 0.92 (0.68-1.16) | 0.06 (-0.02-0.13) | 0.03 (-0.22-0.28) | 0.08 (-0.16-0.32) |
|  | Struggle to make ends meet | 1.39 (1.23-1.55) | 1.11 (0.77-1.46) | 0.02 (-0.08-0.12) | -0.20 (-0.59-0.18) | -0.11 (-0.46-0.23) |
|  | Financial hardship | 1.51 (1.31-1.71) | 0.80 (0.51-1.09) | 0.10 (-0.01-0.21) | 0.15 (-0.14-0.45) | 0.20 (-0.09-0.49) |
| Chronic respiratory disease | Educational level | 1.24 (1.11-1.36) | 0.83 (0.60-1.05) | 0.09 (-0.01-0.18) | 0.12 (-0.10-0.35) | 0.17 (-0.05-0.40) |
|  | Occupational position | 1.24 (1.11-1.36) | 0.89 (0.69-1.09) | 0.03 (-0.05-0.10) | 0.10 (-0.10-0.29) | 0.11 (-0.09-0.31) |
|  | Household income | 1.48 (1.34-1.61) | 0.90 (0.79-1.01) | 0.07 (0.01-0.12) | 0.06 (-0.06-0.18) | 0.10 (-0.01-0.21) |
|  | Health insurance subsidy | 1.48 (1.34-1.63) | 0.93 (0.78-1.07) | 0.06 (-0.01-0.13) | 0.02 (-0.12-0.17) | 0.07 (-0.07-0.22) |
|  | Struggle to make ends meet | 1.46 (1.31-1.60) | 0.87 (0.70-1.05) | 0.10 (0.00-0.19) | 0.05 (-0.12-0.23) | 0.13 (-0.05-0.30) |
|  | Financial hardship | 1.48 (1.31-1.65) | 0.90 (0.72-1.09) | 0.08 (-0.02-0.17) | 0.04 (-0.14-0.22) | 0.10 (-0.09-0.28) |
| Multimorbidity | Educational level | 1.21 (1.09-1.34) | 0.91 (0.52-1.30) | 0.25 (0.09-0.42) | -0.26 (-0.78-0.26) | 0.09 (-0.30-0.48) |
|  | Occupational position | 1.22 (1.09-1.35) | 0.94 (0.56-1.31) | 0.20 (0.06-0.34) | -0.19 (-0.66-0.27) | 0.06 (-0.31-0.44) |
|  | Household income | 1.47 (1.33-1.60) | 0.87 (0.70-1.04) | 0.14 (0.07-0.21) | -0.01 (-0.22-0.19) | 0.13 (-0.04-0.30) |
|  | Health insurance subsidy | 1.48 (1.34-1.63) | 0.82 (0.59-1.06) | 0.14 (0.06-0.23) | 0.05 (-0.20-0.30) | 0.18 (-0.06-0.41) |
|  | Struggle to make ends meet | 1.45 (1.30-1.59) | 0.81 (0.56-1.07) | 0.17 (0.07-0.26) | 0.03 (-0.25-0.31) | 0.19 (-0.07-0.44) |
|  | Financial hardship | 1.48 (1.31-1.65) | 0.79 (0.48-1.09) | 0.18 (0.06-0.30) | 0.05 (-0.27-0.37) | 0.21 (-0.09-0.52) |

Results are from counterfactual mediation models. Multimorbidity indicates having at least two chronic conditions (diabetes, hypertension, cardiovascular disease, respiratory disease). The total effect is Hazards ratio (95% CI) from Cox proportional hazards ratio, adjusted for baseline age, sex, and cohort. Proportion of direct association (95% CI) indicates the association between the exposure and the outcome that includes neither interaction nor mediation. Proportion due to mediation indicates the indirect association of the exposure with the outcome that involves pathways through the mediator. Proportion due to interaction indicates the association of the exposure with the outcomes via interaction with the mediator. Eliminated proportion indicates the proportion of the association that would be eliminated if the mediator were removed (e.g., fixed to 0). Sample size = 3643.

**Supplementary table 6**. Mediation of chronic disease in the association between socioeconomic condition and subsequent prefrailty, Lausanne Cohort 65+

| **Exposure** | **Mediator** | **Total risk**  **HR (95% CI)** | **Proportion of direct association**  **(95% CI)** | **Overall proportion due to mediation**  **(95% CI)** | **Overall proportion due to interaction**  **(95% CI)** | **Overall portion eliminated**  **(95% CI)** |
| --- | --- | --- | --- | --- | --- | --- |
| Obesity | Educational level | 1.22 (1.10-1.34) | 0.57 (0.22-0.92) | 0.41 (0.19-0.64) | 0.03 (-0.38-0.43) | 0.43 (0.08-0.78) |
|  | Occupational position | 1.24 (1.11-1.36) | 0.64 (0.31-0.96) | 0.30 (0.12-0.47) | 0.10 (-0.25-0.46) | 0.36 (0.04-0.69) |
|  | Household income | 1.40 (1.27-1.53) | 0.75 (0.57-0.93) | 0.16 (0.08-0.24) | 0.13 (-0.07-0.33) | 0.25 (0.07-0.43) |
|  | Health insurance subsidy | 1.39 (1.25-1.52) | 0.77 (0.52-1.01) | 0.14 (0.05-0.24) | 0.12 (-0.13-0.37) | 0.23 (-0.01-0.48) |
|  | Struggle to make ends meet | 1.28 (1.16-1.40) | 1.32 (1.19-1.45) | 0.16 (0.06-0.27) | 0.13 (0.03-0.23) | -0.12 (-0.44-0.20) |
|  | Financial hardship | 1.32 (1.16-1.48) | 0.71 (0.31-1.11) | 0.25 (0.07-0.44) | 0.06 (-0.35-0.46) | 0.29 (-0.11-0.69) |
| Diabetes | Educational level | 1.13 (1.02-1.24) | 0.99 (0.87-1.11) | 0.03 (-0.02-0.09) | -0.04 (-0.18-0.11) | 0.01 (-0.11-0.13) |
|  | Occupational position | 1.17 (1.05-1.29) | 1.03 (0.94-1.12) | 0.01 (-0.02-0.04) | -0.07 (-0.18-0.05) | -0.03 (-0.12-0.06) |
|  | Household income | 1.34 (1.22-1.47) | 1.00 (0.96-1.05) | 0.01 (-0.01-0.03) | -0.04 (-0.10-0.02) | 0.00 (-0.05-0.04) |
|  | Health insurance subsidy | 1.33 (1.20-1.46) | 0.98 (0.90-1.05) | 0.02 (-0.02-0.06) | 0.00 (-0.08-0.07) | 0.02 (-0.05-0.10) |
|  | Struggle to make ends meet | 1.29 (1.16-1.42) | 1.02 (0.94-1.10) | 0.01 (-0.03-0.05) | -0.06 (-0.15-0.04) | -0.02 (-0.10-0.06) |
|  | Financial hardship | 1.27 (1.11-1.43) | 1.09 (0.98-1.19) | -0.01 (-0.04-0.02) | -0.11 (-0.24-0.01) | -0.09 (-0.19-0.02) |
| HTA | Educational level | 1.12 (1.01-1.23) | 1.40 (0.58-2.22) | 0.01 (-0.04-0.05) | -0.45 (-1.31-0.41) | -0.40 (-1.22-0.42) |
|  | Occupational position | 1.17 (1.05-1.29) | 0.98 (0.47-1.49) | 0.01 (-0.03-0.04) | 0.01 (-0.51-0.53) | 0.02 (-0.49-0.53) |
|  | Household income | 1.34 (1.22-1.46) | 0.98 (0.74-1.21) | 0.01 (-0.01-0.03) | 0.01 (-0.23-0.26) | 0.02 (-0.21-0.26) |
|  | Health insurance subsidy | 1.33 (1.20-1.46) | 0.95 (0.64-1.27) | 0.02 (-0.02-0.06) | 0.03 (-0.28-0.35) | 0.05 (-0.27-0.36) |
|  | Struggle to make ends meet | 1.29 (1.16-1.41) | 0.75 (0.41-1.10) | 0.03 (-0.02-0.07) | 0.24 (-0.11-0.59) | 0.25 (-0.10-0.59) |
|  | Financial hardship | 1.23 (1.08-1.38) | 1.21 (0.62-1.80) | -0.01 (-0.11-0.09) | -0.25 (-0.85-0.35) | -0.21 (-0.80-0.38) |
| Cardiovascular disease | Educational level | 1.13 (1.02-1.24) | 0.99 (0.54-1.44) | 0.00 (-0.09-0.09) | 0.01 (-0.43-0.45) | 0.01 (-0.44-0.46) |
|  | Occupational position | 1.14 (1.02-1.26) | 1.29 (0.73-1.84) | 0.01 (-0.03-0.06) | -0.34 (-0.93-0.26) | -0.29 (-0.84-0.27) |
|  | Household income | 1.32 (1.20-1.45) | 1.00 (0.81-1.20) | 0.03 (-0.01-0.07) | -0.04 (-0.25-0.18) | 0.00 (-0.20-0.19) |
|  | Health insurance subsidy | 1.32 (1.19-1.45) | 0.93 (0.68-1.18) | 0.05 (-0.02-0.12) | 0.03 (-0.23-0.29) | 0.07 (-0.18-0.32) |
|  | Struggle to make ends meet | 1.26 (1.13-1.39) | 1.09 (0.74-1.45) | 0.03 (-0.07-0.13) | -0.18 (-0.57-0.21) | -0.09 (-0.45-0.26) |
|  | Financial hardship | 1.27 (1.10-1.43) | 0.67 (0.28-1.07) | 0.12 (-0.03-0.27) | 0.28 (-0.11-0.67) | 0.33 (-0.07-0.72) |
| Chronic respiratory disease | Educational level | 1.13 (1.02-1.24) | 0.89 (0.66-1.13) | 0.05 (-0.03-0.14) | 0.07 (-0.16-0.30) | 0.11 (-0.13-0.34) |
|  | Occupational position | 1.17 (1.05-1.28) | 0.95 (0.78-1.12) | 0.00 (-0.05-0.06) | 0.05 (-0.11-0.22) | 0.05 (-0.12-0.22) |
|  | Household income | 1.34 (1.21-1.46) | 0.95 (0.86-1.04) | 0.03 (0.00-0.07) | 0.02 (-0.07-0.12) | 0.05 (-0.04-0.14) |
|  | Health insurance subsidy | 1.33 (1.20-1.46) | 0.98 (0.87-1.09) | 0.03 (-0.02-0.07) | -0.01 (-0.12-0.10) | 0.02 (-0.09-0.13) |
|  | Struggle to make ends meet | 1.28 (1.16-1.40) | 0.92 (0.77-1.08) | 0.06 (-0.02-0.14) | 0.03 (-0.12-0.18) | 0.08 (-0.08-0.23) |
|  | Financial hardship | 1.24 (1.08-1.39) | 0.96 (0.77-1.15) | 0.04 (-0.04-0.12) | 0.00 (-0.18-0.19) | 0.04 (-0.15-0.23) |
| Multimorbidity | Educational level | 1.20 (1.08-1.31) | 0.97 (0.60-1.34) | 0.21 (0.05-0.37) | -0.27 (-0.75-0.21) | 0.03 (-0.34-0.40) |
|  | Occupational position | 1.21 (1.09-1.34) | 0.97 (0.63-1.31) | 0.16 (0.03-0.28) | -0.18 (-0.58-0.23) | 0.03 (-0.31-0.37) |
|  | Household income | 1.38 (1.25-1.51) | 0.89 (0.70-1.07) | 0.14 (0.06-0.22) | -0.04 (-0.26-0.18) | 0.11 (-0.07-0.30) |
|  | Health insurance subsidy | 1.38 (1.25-1.52) | 0.83 (0.57-1.08) | 0.14 (0.05-0.23) | 0.05 (-0.21-0.31) | 0.17 (-0.08-0.43) |
|  | Struggle to make ends meet | 0.04 (-0.25-0.34) | 1.33 (1.20-1.46) | 0.18 (0.08-0.29) | 0.17 (0.05-0.28) | -0.05 (-0.38-0.27) |
|  | Financial hardship | 1.33 (1.17-1.49) | 0.85 (0.46-1.24) | 0.19 (0.03-0.35) | -0.06 (-0.48-0.35) | 0.15 (-0.24-0.54) |

Results are from counterfactual mediation models. Multimorbidity indicates having at least two chronic conditions (diabetes, hypertension, cardiovascular disease, respiratory disease). The total effect is Hazards ratio (95% CI) from Cox proportional hazards ratio, adjusted for baseline age, sex, cohort, smoking, BMI (except model with obesity as mediator), and isolation feeling. Proportion of direct association (95% CI) indicates the association between the exposure and the outcome that includes neither interaction nor mediation. Proportion due to mediation indicates the indirect association of the exposure with the outcome that involves pathways through the mediator. Proportion due to interaction indicates the association of the exposure with the outcomes via interaction with the mediator. Eliminated proportion indicates the proportion of the association that would be eliminated if the mediator were removed (e.g., fixed to 0). Sample size = 3643.

**Supplementary table 7**. Mediation of chronic conditions in the association baseline socioeconomic condition and subsequent frailty, LC65+ cohort, excluding participants with baseline BMI<18.5 and BMI>40

| **Exposure** | **Mediator** | **Total risk**  **HR (95% CI)** | **Proportion of direct association**  **(95% CI)** | **Overall proportion due to mediation**  **(95% CI)** | **Overall proportion due to interaction**  **(95% CI)** | **Overall portion eliminated**  **(95% CI)** |
| --- | --- | --- | --- | --- | --- | --- |
| Obesity | Educational level | 1.44 (1.02-1.86) | 0.26 (-0.37-0.89) | 0.53 (0.13-0.92) | 0.40 (-0.25-1.05) | 0.74 (0.11-1.37) |
|  | Occupational position | 1.43 (0.97-1.89) | 0.36 (-0.27-1.00) | 0.39 (0.05-0.73) | 0.38 (-0.27-1.04) | 0.64 (0.00-1.27) |
|  | Household income | 2.48 (1.74-3.21) | 0.61 (0.39-0.84) | 0.14 (0.06-0.22) | 0.34 (0.10-0.58) | 0.39 (0.16-0.61) |
|  | Health insurance subsidy | 2.26 (1.67-2.84) | 0.60 (0.30-0.90) | 0.15 (0.05-0.26) | 0.33 (0.03-0.64) | 0.40 (0.10-0.70) |
|  | Struggle to make ends meet | 2.54 (1.91-3.16) | 0.78 (0.51-1.05) | 0.10 (0.02-0.19) | 0.16 (-0.11-0.43) | 0.22 (-0.05-0.49) |
|  | Financial hardship | 2.63 (1.82-3.43) | 0.36 (0.02-0.70) | 0.29 (0.13-0.45) | 0.56 (0.23-0.89) | 0.64 (0.30-0.98) |
| Diabetes | Educational level | 1.44 (1.02-1.86) | 0.74 (0.35-1.13) | 0.20 (-0.02-0.42) | 0.11 (-0.31-0.54) | 0.26 (-0.13-0.65) |
|  | Occupational position | 1.41 (0.96-1.85) | 0.66 (0.21-1.12) | 0.20 (-0.04-0.45) | 0.23 (-0.23-0.68) | 0.34 (-0.12-0.79) |
|  | Household income | 2.35 (1.66-3.03) | 0.79 (0.63-0.96) | 0.12 (0.03-0.22) | 0.18 (0.01-0.36) | 0.21 (0.04-0.37) |
|  | Health insurance subsidy | 2.26 (1.69-2.83) | 0.89 (0.68-1.10) | 0.09 (-0.02-0.20) | 0.04 (-0.17-0.25) | 0.11 (-0.10-0.32) |
|  | Struggle to make ends meet | 2.69 (2.00-3.38) | 0.73 (0.51-0.96) | 0.14 (0.02-0.26) | 0.23 (0.01-0.45) | 0.27 (0.04-0.49) |
|  | Financial hardship | 2.70 (1.88-3.52) | 0.72 (0.45-0.98) | 0.13 (-0.01-0.27) | 0.25 (0.00-0.51) | 0.28 (0.02-0.55) |
| Multimorbidity | Educational level | 1.48 (1.04-1.92) | 0.41 (-0.13-0.95) | 0.37 (0.11-0.63) | 0.34 (-0.28-0.96) | 0.59 (0.05-1.13) |
|  | Occupational position | 1.46 (0.98-1.94) | 0.45 (-0.13-1.02) | 0.29 (0.04-0.54) | 0.37 (-0.26-0.99) | 0.55 (-0.02-1.13) |
|  | Household income | 2.56 (1.79-3.33) | 0.43 (0.21-0.65) | 0.21 (0.12-0.31) | 0.54 (0.30-0.78) | 0.57 (0.35-0.79) |
|  | Health insurance subsidy | 2.31 (1.69-2.92) | 0.54 (0.23-0.85) | 0.21 (0.09-0.33) | 0.38 (0.06-0.70) | 0.46 (0.15-0.77) |
|  | Struggle to make ends meet | 2.64 (1.97-3.31) | 0.62 (0.35-0.89) | 0.17 (0.07-0.28) | 0.30 (0.02-0.59) | 0.38 (0.11-0.65) |
|  | Financial hardship | 2.67 (1.87-3.47) | 0.55 (0.20-0.90) | 0.22 (0.08-0.37) | 0.36 (0.01-0.71) | 0.45 (0.10-0.80) |

Results are from counterfactual mediation models. Multimorbidity indicates having at least two chronic conditions (diabetes, hypertension, cardiovascular disease, chronic respiratory disease). The total association is Hazards ratio (95% CI) from Cox proportional hazards ratio, adjusted for baseline age, sex, and cohort. Proportion of direct association (95% CI) indicates the association between the exposure and the outcome that includes neither interaction nor mediation. Proportion due to mediation indicates the indirect association of the exposure with the outcome that involves pathways through the mediator. Proportion due to interaction indicates the association of the exposure with the outcomes via interaction with the mediator. Eliminated proportion indicates the proportion of the association that would be eliminated if the mediator were removed (e.g., fixed to 0). Sample size = 3537.

**Supplementary table 8**. Mediation of chronic conditions in the association baseline socioeconomic condition and subsequent frailty, LC65+ cohort, excluding participants with a mini nutritional assessment score <24 (i.e., at risk of malnutrition or malnutrition)

| **Exposure** | **Mediator** | **Total risk**  **HR (95% CI)** | **Proportion of direct association**  **(95% CI)** | **Overall proportion due to mediation**  **(95% CI)** | **Overall proportion due to interaction**  **(95% CI)** | **Overall portion eliminated**  **(95% CI)** |
| --- | --- | --- | --- | --- | --- | --- |
| Obesity | Educational level | 1.50 (0.97-2.03) | 0.30 (-0.33-0.94) | 0.51 (0.12-0.89) | 0.33 (-0.44-1.10) | 0.70 (0.06-1.33) |
|  | Occupational position | 1.47 (0.89-2.05) | 0.30 (-0.42-1.03) | 0.44 (0.04-0.84) | 0.40 (-0.39-1.19) | 0.70 (-0.03-1.42) |
|  | Household income | 2.19 (1.42-2.95) | 0.44 (0.13-0.76) | 0.21 (0.09-0.34) | 0.49 (0.15-0.83) | 0.56 (0.24-0.87) |
|  | Health insurance subsidy | 2.01 (1.34-2.68) | 0.72 (0.26-1.19) | 0.13 (0.00-0.25) | 0.19 (-0.30-0.68) | 0.28 (-0.19-0.74) |
|  | Struggle to make ends meet | 2.56 (1.78-3.33) | 0.78 (0.42-1.13) | 0.11 (0.00-0.21) | 0.16 (-0.21-0.52) | 0.22 (-0.13-0.58) |
|  | Financial hardship | 2.10 (1.26-2.94) | 0.28 (-0.25-0.81) | 0.29 (0.07-0.51) | 0.60 (0.08-1.13) | 0.72 (0.19-1.25) |
| Diabetes | Educational level | 1.51 (0.99-2.03) | 0.58 (0.09-1.07) | 0.22 (-0.03-0.46) | 0.34 (-0.15-0.82) | 0.42 (-0.07-0.91) |
|  | Occupational position | 1.43 (0.86-1.99) | 0.34 (-0.40-1.07) | 0.33 (-0.05-0.71) | 0.59 (-0.12-1.30) | 0.66 (-0.07-1.40) |
|  | Household income | 2.02 (1.33-2.72) | 0.76 (0.53-0.99) | 0.16 (0.01-0.30) | 0.20 (-0.04-0.44) | 0.24 (0.01-0.47) |
|  | Health insurance subsidy | 2.05 (1.40-2.71) | 0.89 (0.62-1.17) | 0.10 (-0.05-0.25) | 0.02 (-0.26-0.30) | 0.11 (-0.17-0.38) |
|  | Struggle to make ends meet | 2.77 (1.91-3.62) | 0.72 (0.46-0.98) | 0.14 (0.00-0.29) | 0.24 (-0.01-0.50) | 0.28 (0.02-0.54) |
|  | Financial hardship | 2.12 (1.26-2.98) | 0.56 (0.14-0.99) | 0.22 (-0.03-0.46) | 0.38 (-0.02-0.78) | 0.44 (0.01-0.86) |
| Multimorbidity | Educational level | 1.56 (1.00-2.13) | 0.33 (-0.24-0.89) | 0.36 (0.10-0.63) | 0.46 (-0.18-1.11) | 0.67 (0.11-1.24) |
|  | Occupational position | 1.59 (0.94-2.23) | 0.11 (-0.51-0.74) | 0.34 (0.07-0.62) | 0.75 (0.13-1.38) | 0.89 (0.26-1.51) |
|  | Household income | 2.23 (1.45-3.01) | 0.38 (0.07-0.68) | 0.23 (0.11-0.36) | 0.58 (0.25-0.91) | 0.62 (0.32-0.93) |
|  | Health insurance subsidy | 2.03 (1.34-2.72) | 0.62 (0.16-1.08) | 0.16 (0.02-0.30) | 0.29 (-0.19-0.77) | 0.38 (-0.08-0.84) |
|  | Struggle to make ends meet | 2.69 (1.85-3.52) | 0.64 (0.31-0.98) | 0.14 (0.03-0.25) | 0.29 (-0.06-0.63) | 0.36 (0.02-0.69) |
|  | Financial hardship | 2.28 (1.37-3.19) | 0.25 (-0.21-0.71) | 0.33 (0.12-0.53) | 0.64 (0.18-1.10) | 0.75 (0.29-1.21) |

Results are from counterfactual mediation models. Multimorbidity indicates having at least two chronic conditions (diabetes, hypertension, cardiovascular disease, chronic respiratory disease). The total association is Hazards ratio (95% CI) from Cox proportional hazards ratio, adjusted for baseline age, sex, and cohort. Proportion of direct association (95% CI) indicates the association between the exposure and the outcome that includes neither interaction nor mediation. Proportion due to mediation indicates the indirect association of the exposure with the outcome that involves pathways through the mediator. Proportion due to interaction indicates the association of the exposure with the outcomes via interaction with the mediator. Eliminated proportion indicates the proportion of the association that would be eliminated if the mediator were removed (e.g., fixed to 0). Sample size = 3040
